# α-tubulin N-acetyltransferase 1 regulates NCOA4-mediated ferritinophagy and protects cancer cells from ferroptosis

**DOI:** 10.1186/s11658-026-00964-2

**Published:** 2026-06-12

**Authors:** Francesca Romana Pellegrini, Angela Iuzzolino, Cristina De Palma, Francesca Ambrosio, Giulia Fianco, Francesco Davide Naso, Alessio Paone, Serena Rinaldo, Francesca Cutruzzolà, Francesca Degrassi, Flavie Strappazzon, Venturina Stagni, Daniela Trisciuoglio

**Affiliations:** 1 Institute of Molecular Biology and Pathology, National Research Council (CNR) c/o Sapienza University of Rome, Rome, Italy; 2 IRCCS Santa Lucia Foundation, Via del Fosso di Fiorano 64/65, 00143 Rome, Italy; 3 Department of Biochemical Sciences, Sapienza University of Rome, Rome, Italy; 4 Physiopathologie et Génétique du Neurone et du Muscle, UMR5261, U1315, Institut NeuroMyogène, Univ Lyon 1, CNRS, INSERM, 69008 Lyon, France

**Keywords:** α-tubulin N-acetyltransferase 1, Autophagic flux, Microtubules, Reactive oxygen species, Iron metabolism

## Abstract

**Background:**

Microtubules acetylation has emerged as a key regulator of cellular homeostasis, but its roles in autophagy remain understudied. Here, we identify α-tubulin acetyltransferase 1 (ATAT1), the enzyme responsible for α-tubulin K40 acetylation, as a critical regulator of NCOA4-mediated ferritinophagy and iron homeostasis in cancer cells.

**Methods:**

Human cancer cell lines were stably or transiently silenced for ATAT1 expression. Autophagy induction was evaluated by visualizing punctate structures and by analyzing changes in autophagic marker levels. Seahorse and flow cytometry experiments were conducted to study the impact of ATAT1 silencing on cell metabolism. Additionally, analysis of iron homeostasis genes, free iron pool, as well as colocalization of NCO4A and ferritin to autophagosome were analyzed to confirm activation of ferritinophagy. Finally, we treated cells with RSL3 (a ferroptosis inducer) and ferrostatin-1 or chloroquine to understand the connection between ATAT1, autophagy, and ferroptosis-induced cell death. Genetic approaches were used to study the role of NCO4A and K40 acetylation in these pathways.

**Results:**

We show that ATAT1 silencing induces an oxidative stress response accompanied by a functional autophagic flux. Notably, ATAT1-silenced cells exhibited reduced ATP production and oxygen consumption rate compared with control cells, as well as altered mitochondrial dynamics under both normal and stress conditions. Importantly, ATAT1 loss leads to intracellular iron overload by inducing NCOA4-mediated ferritinophagy, which targets the degradation of the iron storage protein ferritin, thus maintaining intracellular iron homeostasis. Activation of ferritinophagy, in turn, renders ATAT1-silenced lung cancer cells more susceptible to ferroptotic cell death. Notably, the key phenotypes observed in ATAT1-silenced cells are absent in cells with non-acetylatable α-tubulin, demonstrating a direct role for the loss of ATAT1 protein on the induction of a ferroptosis vulnerability phenotype.

**Conclusions:**

These findings challenge the traditional view of ATAT1 as a simple microtubule modifier and position this acetyltransferase as a central node in redox, metabolic, and autophagic regulation.

**Graphical abstract:**

**Figure:**
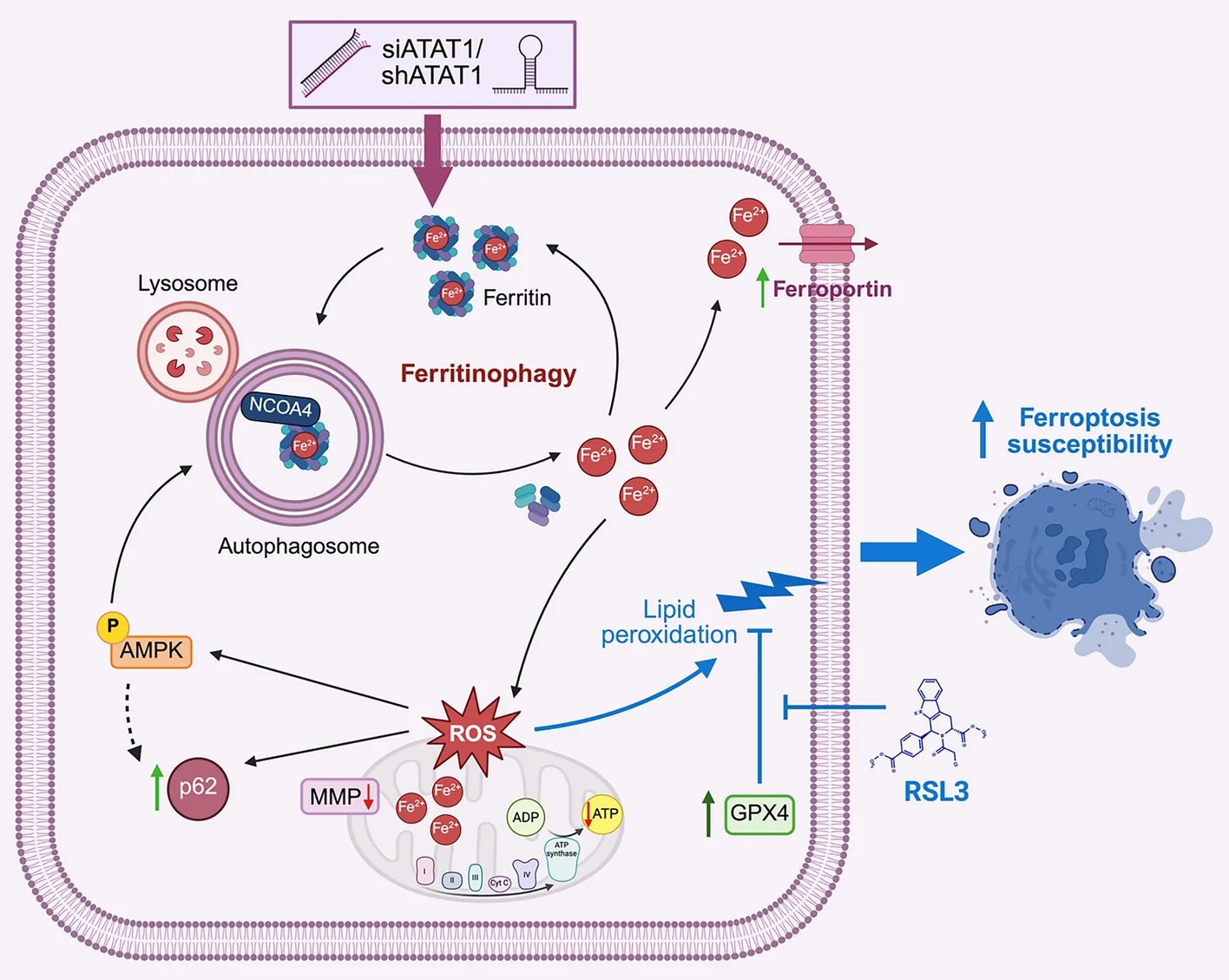

**Supplementary Information:**

The online version contains supplementary material available at 10.1186/s11658-026-00964-2.

## Background

Macroautophagy/autophagy is a fundamental cellular process playing a critical role in the degradation and recycling of cellular components [1]. Autophagy is categorized as either a nonselective bulk degradation system that removes large portions of cellular materials indiscriminately, or a highly selective process, targeting specific cellular components for degradation. In selective autophagy, the cell recognizes and incorporates a particular cargo into autophagosomes for recycling or disposal. Based on the target material to be degraded in the lysosomes, different types of selective autophagy have been described [2]. While some of these specific processes such as mitophagy or endoplasmic reticulum (ER)-phagy are well studied [3, 4], others, such as lipophagy or ferritinophagy, remain poorly understood [5, 6].

Ferritinophagy is an autophagic process mediated by nuclear receptor coactivator 4 (NCOA4) that aims at degrading cytosolic, iron-containing ferritin nanocages [7, 8]. Once ferritin is bound to the cargo receptor NCOA4, it is incorporated into autophagosomes and transported to lysosomes, where the protein is degraded, and iron is released into the cytoplasm for use. Under iron-rich conditions, NCOA4 is eliminated by both proteasomal degradation and basal autophagy, leading to reduced ferritinophagy and higher levels of ferritin-mediated iron storage [6, 9]. On the contrary, under iron-depleted conditions, NCOA4 is stabilized and mediates the autophagosome engulfment of ferritin, facilitating its degradation in the autolysosome and allowing the recycling of the ferritin-bound iron to replenish the cellular labile iron pool [7, 10, 11]. An aberration in the regulatory mechanisms of ferritinophagy has been associated with diverse diseases, including cancer, aging, and neurodegenerative diseases [12]. In tumor biology, ferritinophagy exhibits a dual role contributing to oncogenic metabolic support via iron mobilization while also serving as a mechanism for ferroptosis‑dependent tumor cell elimination [13].

Ferroptosis is an iron-dependent form of regulated cell death defined by an enhanced lipid peroxidation in cell membrane, caused by the iron-dependent oxidation of polyunsaturated fatty acids [14–17]. Recently, ferroptosis has emerged as a promising target for cancer treatment owing to its significant impact on tumor growth and drug resistance [18, 19]. While several studies have emphasized the importance of iron metabolism and ferritinophagy in ferroptosis [20–22], the underlying mechanisms and the activation of the ferritinophagy-dependent ferroptosis pathway remain unclear.

Microtubules (MTs) harboring different tubulin post-translational modifications (PTMs) play a pivotal role in several cellular functions [23–29]. Among PTMs, acetylation of lysine 40 on α-tubulin (hereafter referred to as K40 acetylation) is crucial for numerous cellular processes, including vesicle transport along MTs, cilia formation, cell polarity and migration, intracellular trafficking, and mitochondrial dynamics [29]. Given its importance, alterations of K40 acetylation have been implicated in several neurological diseases [30–34] and in cancer progression [35, 36]. To date, the only known enzyme responsible for K40 acetylation is ATAT1 (α-tubulin acetyltransferase 1) [37–39]. K40 acetylation mediated by ATAT1 can affect different cellular pathways, such as cell motility and invasion [36, 40], mitochondrial movement [41], cell death, and drug resistance [42–44]. K40 acetylation is also linked to autophagy [45–48]. However, only few studies have shown a direct link between K40 acetylation, ATAT1, and the modulation of autophagy [49, 50].

Here, we demonstrated that ATAT1 silencing sensitizes cancer cells to ferroptosis by enhancing NCOA4-mediated ferritinophagy and disrupting iron and oxidative balance in cancer cells. These results reveal new roles for ATAT1 in autophagy regulation and in mitochondrial and iron homeostasis beyond its canonical role as a microtubule modifier.

## Methods

### Cell culture, transfection, and drug treatments

HeLa cells were cultured in high glucose Dulbecco’s modified Eagle’s medium (DMEM) (Euroclone, ECB7501L). A549 and H1299 and the derived cell lines were cultured in RPMI 1640 (Euroclone, ECB9006L).

Media were supplemented with 10% fetal bovine serum (Sial, yourSIAL-FBS-SA), 1% L-glutamine (Euroclone, ECB3000D), 1% penicillin/streptomycin (Euroclone, ECB3001D). All cells (originally obtained by ATCC) were maintained at 37 °C in a humidified incubator with 5% CO_2_. Cells were routinely tested for mycoplasma contamination and were used for no longer than 1 month after thawing.

EGFP-LC3 H1299 and mRFP-EGFP-LC3 H1299 cells were generated as previously described [50]. EGFP-tubulin WT (addgene #38,487) and the EGFP-tubulin K40R vector (addgene #30,488) were used to generate stable H1299 cells, as previously described [51]. mKeima-Red-Mito-7 vector (a kind gift of Dr. G. Cestra, CNR-IBPM), was used to generate Mito-Keima H1299 cells.

H1299 and A549 shATAT1 and shCTR stable cell lines were generated by infection with the lentiviral vectors pLV[shRNA]-Puro-U6 > hATAT1 (VB900122-2317vun) and pLV[shRNA]-Puro-U6 > Scramble shRNA (VB010000-9541pqu, Vector Builder Inc). After infection, cells were selected by the addition of puromycin in the medium culture for 2 weeks and regularly tested for ATAT1 expression and K40 acetylation protein levels.

For transient transfection, cells were transfected with 100 nM siRNA: Negative Control DsiRNA IDT 238932162; siATAT1 or siNCOA4 IDT TriFECTa® RNAi Kit; siATG7 IDT 285851119 or siBECN1, IDT 235851116 (Integrated DNA Technologies) using INTERFERin® (Polyplus, 101,000,028) or Lipofectamine® RNAiMAX Reagent (Invitrogen, 13,778) according to the manufacturer’s instructions for 48 h or 72 h. For EGFP-PRKN experiments, cells were seeded on glass coverslips and transfected the following day with 1 µg EGFP-PRKN plasmid [52] using JetOptimus DNA transfection reagent (Polyplus, 101,000,006) according to the manufacturer’s instructions.

At 24 h from seeding, cells were transfected and treated for 3 h with HBSS (Sigma-Aldriach, H9269) or CQ (Sigma-Aldrich, C6628), starting 45 h from transfection. NH4Cl (Sigma-Aldriach, 09718) was dissolved in water at a stock concentration of 1.43 M. RSL3 treatment and Ferrostatin-1 were dissolved in dimethyl sulfoxide (DMSO) and diluted right before usage at indicated final concentration. Treatments were performed for the last 24 h of culture. Ammonium iron (II) sulfate (Mohr’s salt) and FCCP were used at a final concentration of 100 µM and 10 µM, respectively.

### Immunoblot

Cells were collected by centrifugation and lysed in RIPA buffer supplemented with protease and phosphatase inhibitors cocktails. Between 15 and 30 µg of total proteins were resolved under reducing conditions by sodium dodecyl sulfate polyacrylamide gel electrophoresis (SDS-PAGE) in 5–15% gels, transferred to a nitrocellulose filter (Bio-Rad Laboratories, 1,620,112), blocked with 5% albumin bovine serum (BSA) (Sigma-Aldrich, A4503) or 5% low-fat dry milk, and subjected to immunoblot assay, as previously reported [53].

The following antibodies were used for immunodetection: anti-acetyl-α tubulin antibody (Sigma-Aldrich, MABT868), anti-LC3B (Sigma-Aldrich, L7543), anti-GAPDH (Santa Cruz Biotechnology, sc-32233), anti-SQSTM1/p62 (Santa Cruz Biotechnology, sc-28,359), anti-AMPK (Cell Signaling Technology, #2532), anti-NBR1 (Santa Cruz Biotechnology, sc-130380), anti-phospho-AMPK^Thr172^ (Cell Signaling Technology, #2535), anti-ULK1 (Santa Cruz Biotechnology, sc-390904), anti-phospho-ULK1^Ser555^ (Cell Signaling Technology, #5869), anti-mTOR (Cell Signaling Technology, #4517), anti-phospho-mTOR^Ser2448^ (Cell Signaling Technology, #2971), anti-ATG7 (Cell Signaling Technology, 2631), anti-BECN1 (Cell Signaling Technology, 3738), anti-TFRC/CD71 (Cell Signaling Technology, #13,113), anti-NDP52 (Cell Signaling Technology, #60,732), anti-OPTN (Cell Signaling Technology, #70,928), anti-COXII (Abcam, ab110258), anti-COXIV (Abcam, ab33985), anti-TOMM20 (Santa Cruz Biotechnology, sc-17764), anti-FTL (abcam, ab69090; Cell Signaling Technology, #68,106), anti-FTH1 (Cell Signaling Technology, #4393), anti-NCOA4 (Cell Signaling Technology, #66,849; Bethyl Laboratories, A302-271A).

The following secondary antibodies were used: anti-mouse (Bio-Rad Laboratories, 1,706,515) or anti-rabbit (Bio-Rad Laboratories, 1,706,516) IgG-horseradish peroxidase-conjugated antibodies. Signals were detected by enhanced chemiluminescence (Cyanagen, Westar sun XLS142, Westar Antares XLS063). Densitometric evaluation of band intensity was performed using Image Lab or ImageJ software and values were normalized to loading controls.

### Fluorescence microscopy, live cell time-lapse, colocalization experiments, and relative analyses

For fluorescence microscopy, cells were fixed in 3.7% formaldehyde (Sigma, F1635) in DPBS and permeabilized in 0.2% Triton X-100 (Carlo Erba, 600,471), and DNA was counterstained with 0.05 µg/mL DAPI (4,6-diamidino-2-phenylindole; Sigma-Aldrich, D9542). Images were acquired with NIS Elements 5.4 software. For EGFP-LC3 H1299 cells, the number of green spots per cell was automatically counted, using a previously published CellProfiler pipeline [53] (https://cellprofiler.org/published-pipelines). Cells with more than 15 spots (value calculated as five times the median number of spots in siNEG untreated cells) were considered activated. For mRFP-EGFP-LC3 H1299 cells, the % of red and yellow cells was obtained by manual counting.

DQ-BSA-Red (MedChemExpress, Cat. No.: HY-D2449) was used to determine the lysosomal degradation capacity, according to the manufacturer’s instructions.

MITO-ID® Membrane potential cytotoxicity kit (Enzo, ENZ-51019), FerroOrange (Dojindo Laboratories, F374**)** and BioTracker^TM^Mitochondrial FerroGreen Live Cell Probe (Sigma-Aldrich, SCT262) were used according to the manufacturer’s instructions. Images were then analyzed using an automated pipeline created on CellProfiler software.

For live time-lapse recording, mRFP-EGFP-LC3 H1299 cells (5 × 10^4^/well) were seeded in 2-well µ-slides (Ibidi, 80,286). At 24 h from transfection, the medium was changed to RPMI 1640 without Phenol Red (Euroclone, ECM0505L) and time-lapse observation started. Cells were recorded under a Nikon Eclipse Ti2 microscope with a 60x (1.4 NA) objective, equipped with a CrestOptics X-Light V3 confocal spinning disk; during the whole observation cells were kept in a microscope stage incubator (Okolab) at 37 °C and 5% CO_2_. Images were acquired at 20 min intervals for 50 h. Videos and still images were processed using NIS-Elements AR 5.4. Analysis was performed with the previously mentioned CellProfiler pipeline [53].

For EGFP-PRKN experiments, coverslips were counterstained with DAPI, acquired, and cells with or without localized spots of EGFP-PRKN on mitochondria were counted manually.

All samples described above were observed under a Nikon Eclipse Ti2 microscope with a 60× (1.4 NA) objective, equipped with a CrestOptics X-Light V3 confocal spinning disk and a back illuminated Kinetix sCMOS camera, or under an Olympus AX70 microscope using a 100 × /1.35 NA objective.

For NCOA4 and LC3 colocalization experiments, 1 × 10^5^ H1299 cells were seeded on glass coverslips and transfected the following day with 1 µg HA-LC3B plasmid [54, 55] using polyethylenimine (PEI, Tebu-Bio, 23,966–1) according to the manufacturer’s instructions. At 6 h from HA-LC3B transfection, cells were transfected with 100 nM siATAT1 using Lipofectamine® RNAiMAX (ThermoFisher Scientific, 13,778,075) according to the manufacturer’s instructions. Coverslips were processed for immunofluorescence using the primary antibodies anti-NCOA4 (Bethyl laboratories, A302-272A) and anti-HA tag (Santa Cruz Biotechnology, sc-7392) and the following secondary antibodies: Alexa Fluor 546 donkey anti-mouse IgG (H + L) (Invitrogen, A10036) and Alexa Fluor 488 donkey anti-rabbit IgG (H + L) (Invitrogen, A21206). For LC3, Ferritin and LAMP2 colocalization experiments, 1 × 10^5^ H1299 EGFP-LC3 cells were seeded on glass coverslips and transfected the following day with 100 nM siATAT1 using Lipofectamine® RNAiMAX, according to the manufacturer’s instructions. Coverslips were processed for immunofluorescence using the primary antibodies anti-Ferritin (Invitrogen, MA532244) and anti-LAMP2 (Santa Cruz Biotechnology, sc-18822) and the following secondary antibodies: Cy^TM^3 AffiniPure® Donkey Anti-Rabbit IgG (H + L) (Jackson ImmunoResearch, 711–165-152) and Alexa Fluor® 647 AffiniPure® Donkey Anti-Mouse IgG (H + L) (Jackson ImmunoResearch, 715–605-150). DNA was counterstained with 1 µg/ mL Hoechst 33,258 solution (Sigma Aldrich, 94,403). Confocal images were acquired in 1024 × 1024 mode with a Nikon Ti-E microscope equipped with the C2 laser scanning confocal microscope with a 100 × objective using the NIS-Elements 5.4 software or with a Nikon Eclipse Ti2 microscope with a 60x (1.4 NA) objective, equipped with a CrestOptics X-Light V3 confocal spinning disk and a back illuminated Kinetix sCMOS camera. Images were then analyzed using the ComDet plugin of Fiji-ImageJ.

### RT-qPCR

For RNA extraction, 0,4 µg of total RNA isolated by TRIzol reagent (Invitrogen, 15,596,026) was retrotranscribed with yourSial cDNA Synthesis Kit (Sial, SIAL-ycDNA) according to standard procedures. Approximately 10 ng of cDNA were employed to quantify the transcripts by real time RT-qPCR using Luna® Universal qPCR Master Mix (New England Biolabs, #M3003) and gene-specific primers, listed below. RT-qPCR was performed using the QuantStudio^TM^ 3 Real-Time PCR System (Applied Biosystem™, A28567). Relative quantities were calculated, normalizing for ACTB. Mean values and standard deviations of relative quantities were generated from at least three biological replicates, each including three technical replicates.

To quantify the relative mtDNA content, 3 × 10^5^ cells were seeded, transfected when required, and collected. Total DNA was then extracted using the QIAamp DNA Micro Kit (QIAGEN, 56,304). PCR was performed with Power Up SYBR Green Master Mix (Applied Biosystems/Thermo Fisher Scientific, A25741) on a LightCycler 480 thermo-cycler (Roche). mtDNA/nuclear DNA (nDNA) ratio was calculated with the ΔΔCt method, after normalization of the MT-COXII gene with GAPDH used as the housekeeping gene. The Ct of c/n DNA samples for each experiment was the mean of at least two technical replicate values of Ct for each sample.

The following primer sequences were used:

p62-FW 5’–CCA TTG CGG AGC CTC ATC TC–3’.

p62-RV 5’–GTC CCC GTC CTC ATC CTT TC–3’.

Actin-FW 5’–GAT TCC TAT GTG GGC GAC GA–3’.

Actin-RV 5’–CAC AGG ACT CCA TGC CCA G–3’.

MT-COXII-FW 5’–GTCCTGTATGCCCTTTTCCTAACACTC–3’

MT-COXII-RV 5’–GACCTCGTCTGTTATGTAAAGGATGCG–3’

GAPDH-FW 5’–TTC AAC AGC GAC ACC CAC TC–3’

GAPDH-RV 5’–CGC CAG ACC CTG CAC TTT TT–3’

FTL-FW 5’–TAC GAG CGT CTC CTG AAG ATG C–3’

FTL-RV 5’–GGT TCA GCT TTT TCT CCA GGG C–3’

FTH1 FW 5’–TGA AGC TGC AGA ACC AAC GAG G–3’

FTH1 RV 5’–GCA CAC TCC ATT GCA TTC AGC C–3’

FTMT FW 5’–GCT CTA TGC GTC CTA CGT GTA C–3’

FTMT RV 5’–TGG TTC TGC AGC CTC ATC AGC T–3’

TFRC FW 5’–ATC GGT TGG TGC CAC TGA ATG G–3’

TFRC RV 5’–ACA ACA GTG GGC TGG CAG AAA C–3’

SLC40A1 FW 5’–GAG ACA AGT CCT GAA TCT GTG CC–3’

SLC40A1 RV 5’–TTC TTG CAG CAA CTG TGT CAC AG–3’

NCOA4 FW 5’–GCT TGC TAT TGG TGG AGT TCT CC–3’

NCOA4 RV 5’–GCC ATA CCT CAC GGC TTC TAA G–3’

### Flow cytometry

For ROS production analysis, 1 × 10^5^ cells were seeded, transfected the next day and collected 48 h later. H_2_O_2_ (5 mM) added 30 min before harvesting was used as positive control. Samples were collected, washed with DPBS (Euroclone, ECB4004L), stained in 10 µM DHE (dihydroethidium; Sigma-Aldrich, D7008) in medium without serum for 30 min and immediately analyzed.

Mitochondrial membrane polarization (MMP) was measured with the MITO-ID® Membrane potential cytotoxicity kit (Enzo, ENZ-51019), as previously described and immediately analyzed.

For lipid peroxidation and AnnexinV-FITC/PI staining analyses, 1.5 × 10^5^ cells were seeded, treated the next day with 50 nM RSL3 and/or 1 µM Fer-1 for 24 h and collected 24 h later. Samples were collected, washed with DPBS, and stained using the Lipid Peroxidation Sensor BODIPY^TM^ 581/591 C11 (Invitrogen^TM^, D3861) according to the manufacturer’s instructions. For cell death, AnnexinV-FITC/PI staining was performed as previously reported [53].

For cell death analysis in H1299 WT TUBA and TUBA^K40R^ cultures, 1.5 × 10^5^ cells were seeded, treated the next day with 50 nM RSL3 and collected 24 h later. Samples were collected, fixed in 70% ethanol and stained with propidium iodide for 30 min. Sub-G1 population, representative of dead cells, was analyzed.

All cytofluorimetric data were acquired using either the BD FACSCalibur cytofluorimeter or the Beckman coulter CytoFLEX SRT cell sorter. Analyses were performed using either Flowing Software program (2.5.1) or Kaluza software.

### Seahorse assay

Cellular oxygen consumption rate (OCR) and extracellular acidification rate (ECAR) were detected using XF Cell Mito Stress Test (Agilent) measured by the extracellular flux analyzer XFe96 (Seahorse Bioscience, Houston, TX, USA) in the HypACB facility at Sapienza University. H1299 transiently and stably silenced cells (see above) were cultured on XFe colture 96-wells miniplates for 24 h (6 × 10^4^ cells/well). The sensor cartridge for XFe analyzer was hydrated in a 37 °C non-CO_2_ incubator a day before the experiment. Cell Mito Stress Test Kit was used (Kit-103015-100) and, according to the manufacturer instructions, stressors concentrations were optimized and added as follows: 1 µM oligomycin as complex V inhibitor, 1.5 µM FCCP (uncoupler agent) and 0.5 µM rotenone/antimycin A (inhibiotrs of complex I and III). During sensor calibration, cells were incubated in a 37 °C non-CO_2_ incubator in 180 µl assay medium (XF base medium supplemented with 10 µM glucose, 10 mM pyruvate, and 2 mM L-glutamine at pH 7.4, was used to wash the cells and replace the growth medium).

### Statistical analysis

Experiments were replicated at least three times, unless specified, and data are expressed as mean ± standard error (SEM), unless otherwise indicated.

Differences between two groups were analyzed by two-sided unpaired Student’s *t* test. Comparisons between multiple groups were performed using the two-ways analysis of variance (ANOVA) followed by multiple comparison post-hoc test. Data were analyzed using the GraphPad Prism software. Values of significance: * = *p* < 0.05, ** = *p* < 0.01, *** = *p* < 0.001, **** = *p* < 0.0001.

## Results

### ATAT1 silencing leads to autophagic flux activation in cancer cells

**Fig. 1:**
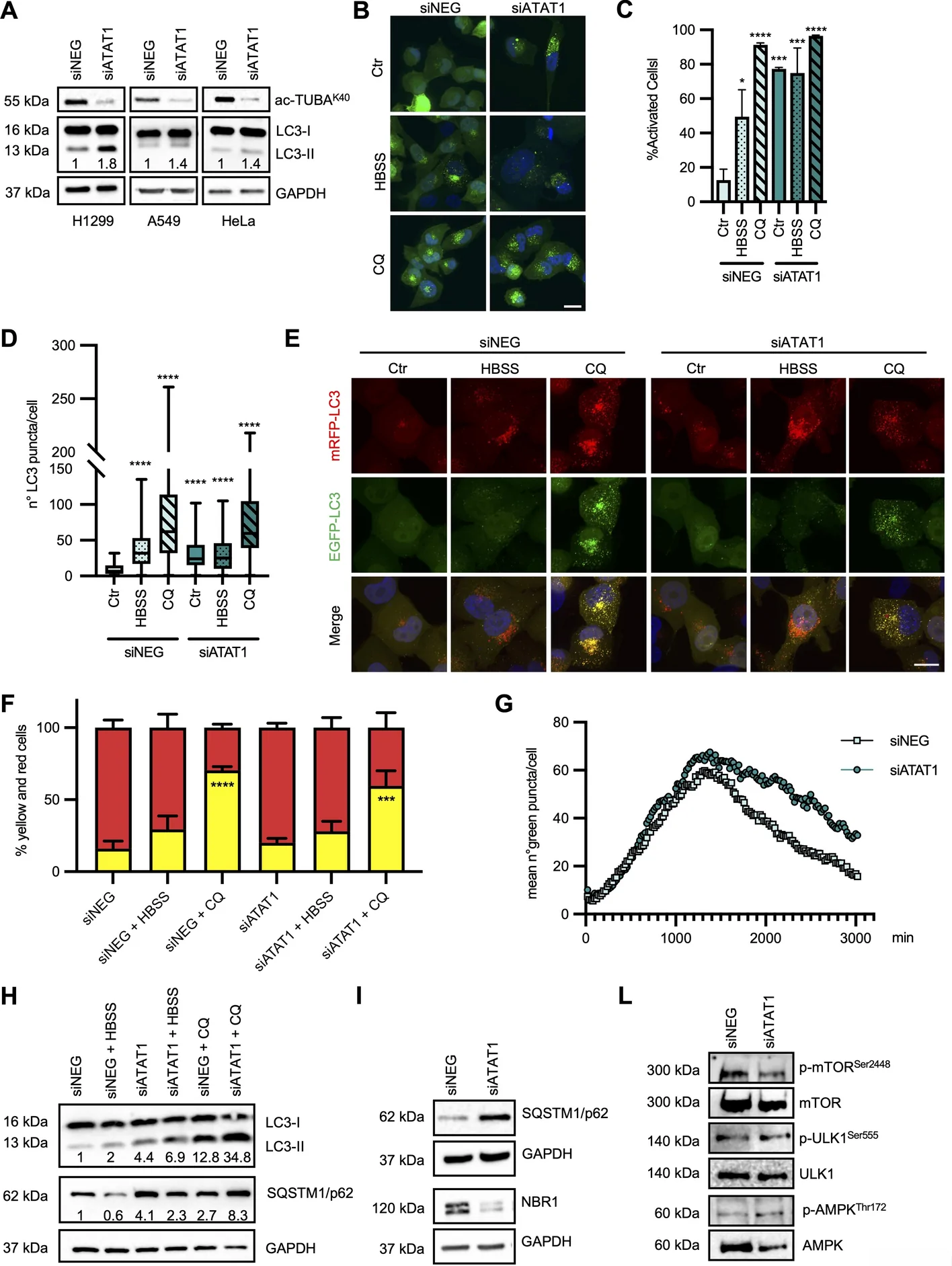
ATAT1 silencing promotes autophagic flux. **A** Immunoblot analysis of LC3 and K40 acetylation of TUBA (ac-TUBA^K40^) in the indicated cancer cells transfected with a control siRNA (siNEG) or a siRNA against ATAT1 (siATAT1). **B** Representative images of EGFP-LC3 H1299 cells transfected with siNEG or siATAT1 and treated with HBSS or 25 µM CQ for 3 h. Scale bar: 20 μm. **C** Quantification of the percentage of activated cells (> 15 EGFP-LC3 spots/cell), in cells treated as in (B). Data are presented as mean ± SEM (*n* = 3) and analyzed using an ordinary one-way ANOVA followed by Fisher’s post-hoc test. *p < 0.05, ***p < 0.001, ****p < 0.0001 versus siNEG. **D** Quantification of the number of EGFP-LC3 puncta/cell, in cells treated as in (**B**). Data are presented as box + min. to max. whisker (*n* = 3) and analyzed using a nonparametric Kruskal–Wallis test followed by Dunn’s post-hoc test. ****p < 0.0001 versus siNEG. **E** Representative images of mRFP-EGFP-LC3 H1299 cells transfected and treated as in (**B**). Scale bar: 20 µm. **F** Quantification of the percentage of cells showing mRFP (red, autolysosomes) or mRFP-EGFP (yellow, autophagosomes) dots for the experiments shown in (**E**). Data are presented as mean ± SEM (*n* = 3) and analyzed using a two-way ANOVA followed by Fisher’s LSD post-hoc test. ***p < 0.001, ****p < 0.0001. **G** Quantification of the mean number of EGFP-LC3 puncta/cell extracted from time-lapse imaging (> 100 cells/time frame) of mRFP-EGFP-LC3 H1299 cells transfected with siNEG or siATAT1. Images were acquired at 20 min intervals for 50 h, starting 24 h post-transfection. H Immunoblot analysis of LC3 and SQSTM1/p62 in H1299 cells transfected with siNEG or siATAT1 and treated with HBSS or CQ as indicated in (B). I Immunoblot analysis of autophagic cargos NBR1 and SQSTM1/p62 in H1299 transfected with siNEG or siATAT1. (L) Immunoblot analysis of p-mTOR^(ser2448)^, total mTOR, p-AMPK^(Thr172)^, total AMPK and p-ULK1.^(ser555)^, total ULK1 in H1299 transfected with siNEG or siATAT1

To ascertain the relationship between K40 acetylation and autophagy, we examined the effects of ATAT1 silencing across diverse tumor cell lines. Firstly, we analyzed changes in the autophagosomal marker microtubule-associated protein 1 light chain 3 (LC3), in lung (H1299, A549), and cervical (HeLa) cancer cells transfected with a siRNA targeting ATAT1 (sequence 13.2, hereafter referred to as siATAT1) or an untargeted siRNA (siNEG). Since the antibodies tested against ATAT1 did not work in our hands, the efficacy of ATAT1 silencing was assessed by western blot analysis using an antibody directed against K40 acetylated α-tubulin (ac-TUBA^K40^), the ATAT1-dependent modification. As shown in Fig. 1A, ATAT1 silencing effectively reduced K40 acetylation levels compared with controls and notably increased the levels of the lipidated form of LC3 (LC3-II) across all cell lines, though with varying efficiency. A different siATAT1 sequence (13.3) was also tested in H1299 cells, showing a comparable efficacy to 13.2 siATAT1 RNA in lowering ATAT1 mRNA by RT-qPCR and reducing ac-TUB^K40^ levels by immunoblot (Supplementary Fig. S1A, B). Similarly, LC3-II accumulation and K40 acetylation reduction were also observed in H1299 and A549 lung cancer cells stably silenced for ATAT1 using a shRNA lentiviral approach (Supplementary Fig. S1C, D).

Next, we analyzed autophagosome formation and autophagic flux by fluorescence microscopy [56]. To this aim, we used H1299 cells stably expressing EGFP-LC3 with and without the autophagy inducer Hank’s Buffer Salt Solution (HBSS) and the late-stage autophagy inhibitor chloroquine (CQ) following ATAT1 silencing. ATAT1-silenced cells showed a redistribution of EGFP-LC3 into cytoplasmic puncta, significantly elevated frequencies of autophagy activated cells and an increased puncta number per cell compared with siNEG cells (Fig. 1B–D). As expected, CQ and HBSS exposure led to an increase in both the percentage of activated cells and puncta number per cell in both control and in ATAT1-silenced cells (Fig. 1B–D).

To study the functionality of the autophagic flux in ATAT1-silenced cells, we utilized H1299 cells stably expressing the tandem fluorescent-labeled mRFP-EGFP-LC3 reporter, which allows to distinguish autophagosomes (yellow signal) from autolysosomes (red signal) [56]. By using this approach, we demonstrated that ATAT1 silencing induces complete autophagosome maturation into autolysosomes in most of activated cells, both under basal conditions and in response to HBSS. As expected, the majority of CQ treated cells were characterized by an impairment in the autophagosome-lysosome fusion both in control and in ATAT1-silenced cells (Fig. 1E). In line with this observation, quantitative analysis shows a similar trend between siNEG and siATAT1 cells across the different conditions analyzed, confirming the presence of a complete autophagic flux (Fig. 1F). DQ‑BSA-Red was also used to detect lysosomal activity in siNEG and siATAT1 cells. As shown in Supplementary Fig. S1E, DQ-BSA was efficiently cleaved in a similar fashion in siNEG and siATAT1 cells under basal conditions, while treatment with CQ or with Bafilomycin A1 (Baf), which prevents the acidification of lysosomes, caused a significant reduction in DQ-BSA-related fluorescence in both cell types, confirming that ATAT1 silencing did not inhibit lysosomal degradation capacity of H1299 cells.

These results were also corroborated by a time lapse video recording analysis of H1299 mRFP-EGFP-LC3 cells following ATAT1 silencing. In both siNEG (Supplementary Video S1) and siATAT1 cells (Supplementary Video S2), there was an initial accumulation of EGFP spots that decreased along time as EGFP signal got quenched due to lysosome fusion. EGFP spot quantification from time-lapse imaging shows that, although the flux was found to be functional, there is also a slowdown in the flux of ATAT1-silenced cells. (Fig. 1G). This aligns with previous studies showing that reduced microtubule acetylation leads to disorganized distribution of autophagosomes and impairs transport along microtubule tracks [47, 48].

To corroborate the activation of autophagy by ATAT1 silencing, we analyzed the modulation of LC3-II and the autophagy receptor protein sequestosome 1 (SQSTM1/p62) in H1299 (Fig. 1H, quantification in Supplementary Fig. S1F,G) and HeLa (Supplementary Fig. S1H) cells in response to HBSS and CQ [56]. Immunoblot analyses corroborated the activation of autophagy in ATAT1 silenced cells by showing the accumulation of LC3-II in H1299 and HeLa cells in basal conditions and in response to HBSS. As expected, CQ treatment caused an accumulation of LC3B-II. This effect was more evident in ATAT1 silenced cells exposed to CQ compared with siNEG controls, demonstrating that ATAT1 silencing induces a complete autophagic flux.

Unexpectedly, the levels of the autophagy receptor protein SQSTM1/p62 significantly increased in siATAT1 cells under basal conditions compared with siNEG controls in both H1299 and HeLa cells. In accordance with a functional activation of the autophagic flux in siATAT1 cells, SQSTM1/p62 was almost completely degraded by autophagy in siATAT1 HBSS-starved cells (Fig. 1H and Supplementary Fig. S1F,G for quantification), suggesting that ATAT1 regulates SQSTM1/p62 expression in basal conditions independently from autophagy. Western Blot of LC3-II and SQSTM1/p62 in H1299 shATAT1 and shCTR cells under basal conditions and in HBSS or CQ-treated conditions confirm the results obtained in transiently silenced cells (Supplementary Fig. S1I). In contrast to p62/SQSTM1, the protein levels of NBR1 autophagy cargo receptor (NBR1) were significantly reduced upon ATAT1 silencing (Fig. 1I and Supplementary Fig. S2A,B for quantification).

Next, we explored whether Unc-51-like kinase 1 (ULK1)/mTOR complex 1 (mTOR)/ AMP-activated protein kinase (AMPK) ULK/mTOR signaling pathways play an important role in ATAT1 -induced autophagy. As depicted in Fig. 1L, we found that ATAT1-silenced cells exhibited an increased activation of AMPK through Thr172 phosphorylation, a decreased mTOR phosphorylation at Ser2448, and a concomitant increase in ULK1 Ser 555-phosphorylation (Fig. 1L and Supplementary Fig. S1C–E for quantification).

To gain deeper insight into the role of ATAT1 in the autophagic process, we evaluated the involvement of the beclin-1 (BECN1)-dependent, or the autophagy related 7 (ATG7)-dependent pathways by a sequential siRNA protocol to target both ATAT1 and autophagic genes. While siBECN1 only attenuated the LC3-II rise induced by siATAT1 (Supplementary Fig. S2F), siATG7 effectively abrogated siATAT1 impact on autophagy (Supplementary Fig. S2G). Moreover, siATAT1 did not significantly modify the level of either BECN1 or ATG7. These findings indicate that siATAT1 is likely to rely on the ATG7-dependent, noncanonical form of autophagy that bypasses the BECN1-dependent formation of autophagosome precursors.

### Autophagy activation in ATAT1-silenced cells stems from elevated ROS levels

**Fig. 2:**
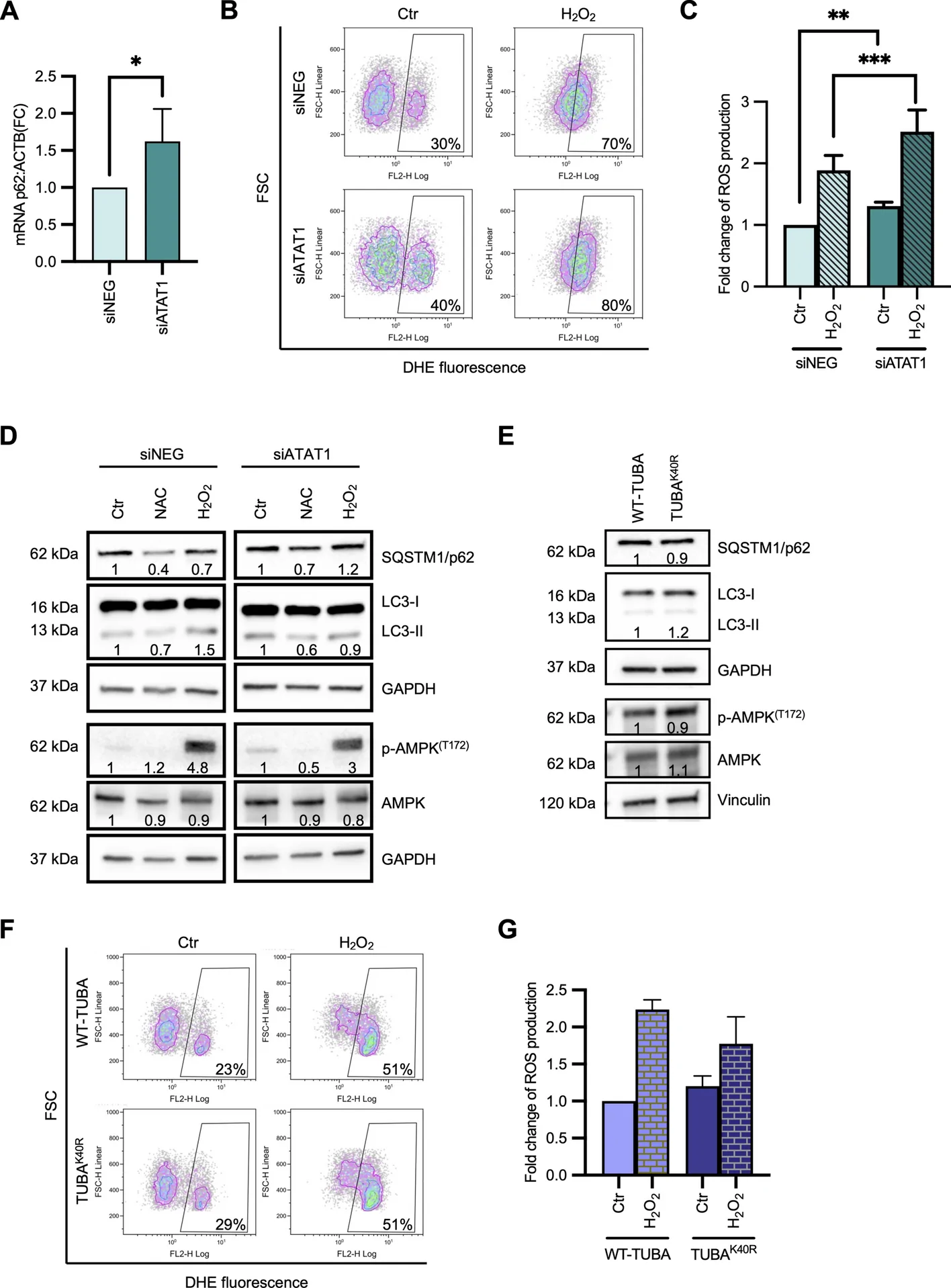
ATAT1 silencing increases oxidative damage. **A** qRT-PCR of SQSTM1/p62 mRNA in H1299 cells transfected with siNEG or siATAT1. SQSTM1/p62 mRNA was normalized over ACTNB (actin beta). Data are presented as fold change ± SEM (*n* = 6) and were analyzed using a Mann–Whitney *U* test. *p < 0.05. **B** Representative density plots and **C** quantification of DHE-positive H1299 cells transfected with siNEG or siATAT1 alone or in combination with 5 mM H_2_O_2_ for 30 min. Data are presented as fold change ± SEM (*n* = 4). **p < 0.01, ***p < 0.001. **D** Immunoblot analysis of LC3 conversion, ac-TUBA^K40^, SQSTM1/p62, p-AMPK^(Thr172)^, and total AMPK in H1299 cells transfected with siNEG or siATAT1 for 48 h and treated with 5 mM NAC for 42 h or 5 mM H_2_O_2_ for 30 min. **E** Immunoblot analysis of LC3 conversion, ac-TUBA^K40^, SQSTM1/p62, p-AMPK^(Thr172)^, and total AMPK in H1299 cells expressing WT-TUBA or the mutant form TUBA^K40R^. Numbers on immunoblots report the densitometric values of LC3-II and SQSTM1/p62 band intensity normalized over GAPDH and of p-AMPK^(Thr172)^ over total AMPK. **F** Representative density plots and **G** quantification of DHE-positive H1299 cells expressing WT-TUBA or the mutant form TUBA^K40R^ alone or in combination with 5 mM H_2_O_2_ for 30 min. Data are presented as fold change ± SEM (*n* = 6)

Since SQSTM1/p62 is a stress response protein strongly induced both at the mRNA and protein levels by different stress, including oxidative stress [57–59], we moved to examine the relationship between ATAT1 and cellular redox status. First, we investigated the mechanism of SQSTM1/p62 regulation by measuring SQSTM1/p62 mRNA levels through quantitative PCR. Interestingly, ATAT1-silenced cells exhibited approximately two-fold higher SQSTM1/p62 mRNA levels than siNEG cells (Fig. 2A), confirming that ATAT1 regulates SQSTM1/p62 expression in basal conditions independently from autophagy. Then, we quantified intracellular reactive oxygen species (ROS) levels both under basal conditions and in response to hydrogen peroxide (H_2_O_2_), a well-established inducer of oxidative stress. As shown in Fig. 2B, C, ATAT1 silencing is associated with an increase in intracellular ROS levels over those observed in siNEG cells, both at baseline and in response to H_2_O_2_.

To determine the interplay between oxidative stress and autophagy following ATAT1 downregulation, we treated cells with the antioxidant N-acetyl-l-cysteine (NAC) or challenged cells with H_2_O_2._ Under basal conditions, NAC treatment reduced LC3 lipidation and SQSTM1/p62 accumulation in siATAT1 cells. Furthermore, NAC treatment reduced AMPK phosphorylation in ATAT1-silenced cells, suggesting ROS involvement in ATAT1-induced autophagic flux (Fig. 2D and Supplementary Fig. S2H–L for quantification). H_2_O_2_ treatment activated the AMPK pathway in siNEG cells, which in turn increased LC3 lipidation. However, H_2_O_2_ treatment did not further modify LC3 lipidation or SQSTM1/p62 levels in ATAT1-silenced cells (Fig. 2D and Supplementary Fig. S2H–L for quantification), suggesting that autophagy is fully activated under basal conditions in siATAT1 cells.

To distinguish the role of K40 acetylation and ATAT1 presence in these pathways, we generated H1299 cells stably expressing either wild-type α-tubulin (WT-TUBA) or a non-acetylatable α-tubulin mutant (TUBA^K40R^) [51]. Analysis of LC3-II and SQSTM1/p62 levels under basal conditions and in response to HBSS or CQ showed no significant differences in TUBA^K40R^ mutated cells compared with WT-TUBA cells (Supplementary Fig. S2M). Similarly, no significant differences in AMPK phosphorylation status (Fig. 2E and quantification in Supplementary Fig. S2N) and ROS levels were observed between WT-TUBA and TUBA^K40R^ cells under basal conditions or when challenged with H_2_O_2_ (Fig. 2F, G).

Overall, these results demonstrate that ATAT1 loss is associated with oxidative stress, which in turn leads to autophagy activation.

### ATAT1 silencing impairs mitochondrial respiration and causes mitochondrial damage without triggering mitophagy

**Fig. 3:**
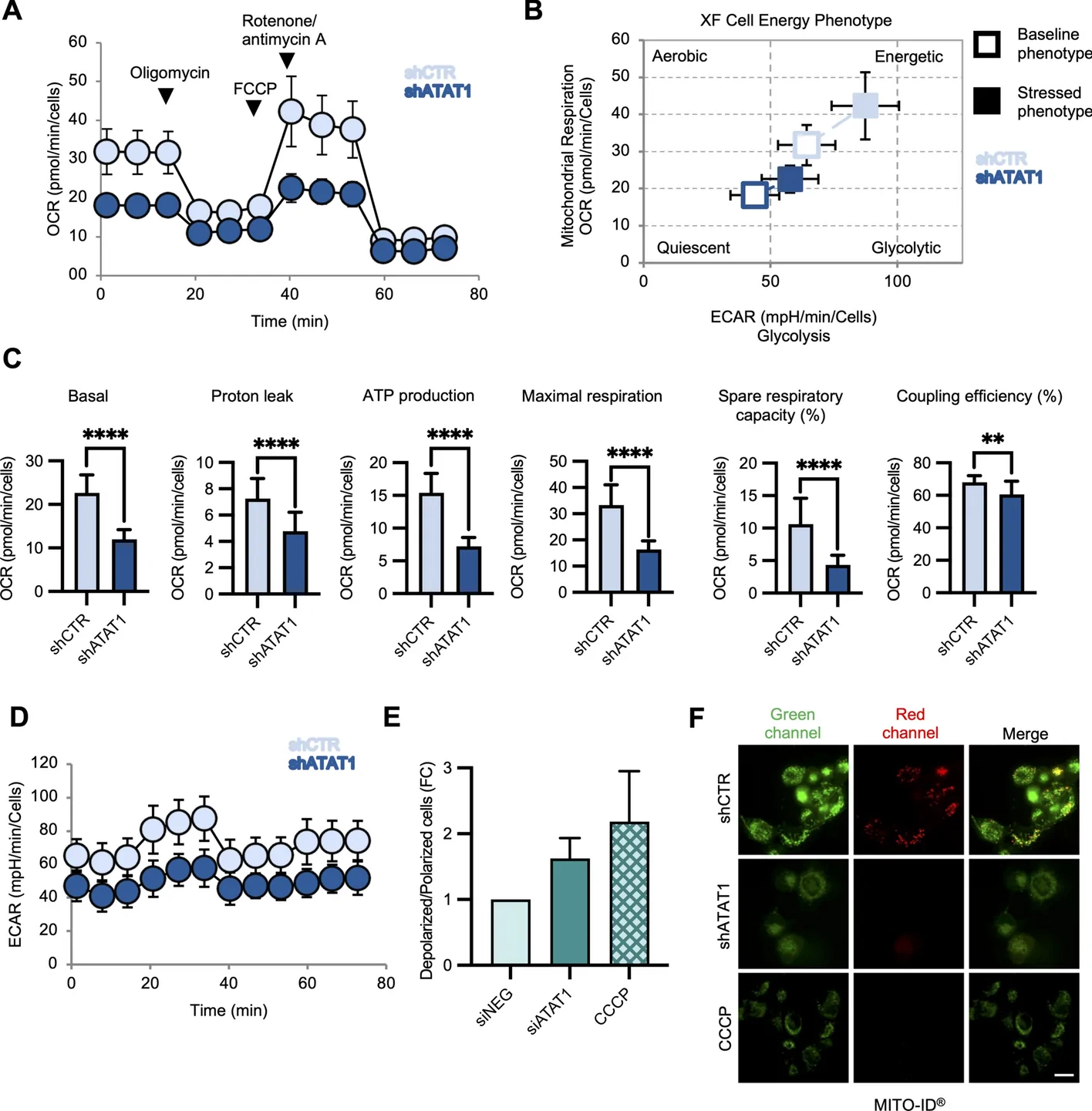
ATAT1 silencing induces a reduction in oxidative phosphorylation (OXPHOS). **A, C** Oxygen consumption rate (OCR) levels over time in H1299 cells stably transfected with shRNA targeting ATAT1 (shATAT1) or with scramble shRNA (shCTR). The measured parameters after stressors administration (arrowheads) are reported, namely: i) oligomycin to target ATP production, which leads to the apparent switch-off of the respiratory chain as compensatory response; the possible residual OCR is due to proton leak, which in principle, may discharge the potential; ii) FCCP, as an uncoupler, to evaluate the capability of the respiratory chain to compensate the insult and to try to keep a proper membrane potential by increasing the OCR; iii) rotenine/antimycin A to definitively switch-off the respiratory chain, and consequently the oxygen consumption carried out by complex IV. Data are presented as mean ± SD (*n* = 3) and were analyzed using a nonparametric Mann–Whitney *U* test. **p < 0.01, ***p < 0.001, ****p < 0.0001. **B** Energetic profile of H1299 cells treated as in (**A**), before (hollow symbol) or after (filled symbol) drug administration. **D** Extra cellular acidification rate (ECAR) levels over time in H1299 cells treated as in (**A**). **E** Quantification of the ratio between cells with a loss in mitochondrial membrane polarization and cells with a fully polarized membrane in H1299 cells transfected with siNEG or siATAT1 and cells treated with 10 µM CCCP for 1 h. Cells were stained using the MITO-ID kit and analyzed by cytofluorimetry. Data are presented as mean ± SEM (*n* = 2). **F** Representative images of H1299 cells stably transfected with shRNA targeting ATAT1 (shATAT1) or with scramble shRNA (shCTR) and cells treated with CCCP for 1 h. Cells were stained using the MITO-ID kit and imaged under a spinning disk microscope. Scale bar: 20 µm

Given AMPK’s role in cellular energy metabolism and mitochondrial adenosine triphosphate (ATP) production [60], we investigated ATAT1 influence on energy metabolism. Using the Seahorse assay, we measured oxygen consumption rate (OCR) and extracellular acidification rate (ECAR) as readouts of mitochondrial respiration and glycolysis in control (shCTR) and ATAT1 (shATAT1) stably silenced cells. Overall, decreased mitochondrial functions (Fig. 3A, C) and a slight reduction in ECAR (Fig. 3D) were observed in ATAT1-silenced cells compared with control ones. Bioenergetics of ATAT1-silenced cells showed a lower energetic profile which is poorly responsive to Mito Stress Kit stressor, as compared with control cells that are characterized by a high energetic status under both basal and stressed conditions (Fig. 3B). The inability to compensate carbonyl cyanide 4-(trifluoromethoxy) phenylhydrazone (FCCP) treatment via OCR increase could be indicative of different mitochondrial membrane potential between the shATAT1 and shCTR cells, which is not apparently dependent on uncoupling events, since the proton leak is even lower in shATAT1 than shCTR.

These experiments lead us to hypothesize that ATAT1 silencing could cause mitochondrial dysfunction and activate mitophagy in order to selectively degrade damaged mitochondria via parkin RBR E3 ubiquitin protein ligase (PRKN)-dependent mitophagy [61]. In line with previous results (Fig. 3A–D), we found that ATAT1-silenced cells showed compromised mitochondrial membrane potential (MMP) (Fig. 3E, F). GFP-tagged Parkin translocation studies demonstrated no significant changes in the percentage of cells recruiting Parkin to mitochondria in response to ATAT1 silencing in both untreated and FCCP-treated cells (Supplementary Fig. S3A,B). To confirm these observations, we generated H1299 cells stably expressing the mKeima-Red-Mito-7 reporter, allowing mitophagy detection [62]. While FCCP treatment induced mitophagy in both control and ATAT1-silenced cells, untreated ATAT1-silenced cells showed no significant mitophagy activation compared with siNEG cells. (Supplementary Fig. S3C).

Furthermore, we observed no changes in expression of key mitophagy markers, including CALCOCO2/NDP52, optineurin (OPTN), cytochrome c oxidase subunits (COX II and COX IV), and TOMM20 (Supplementary Fig. S3D) nor in the ratio of mitochondrial DNA to genomic DNA (Supplementary Fig. S3E,F) in both ATAT1 transiently and stably silenced cells. These findings indicate that ATAT1-dependent autophagy is not a selective form of mitophagy in spite of ATAT1 silenced cells displaying a marked mitochondrial dysfunction.

### ATAT1 silencing alters iron homeostasis and induces ferritinophagy

**Fig. 4:**
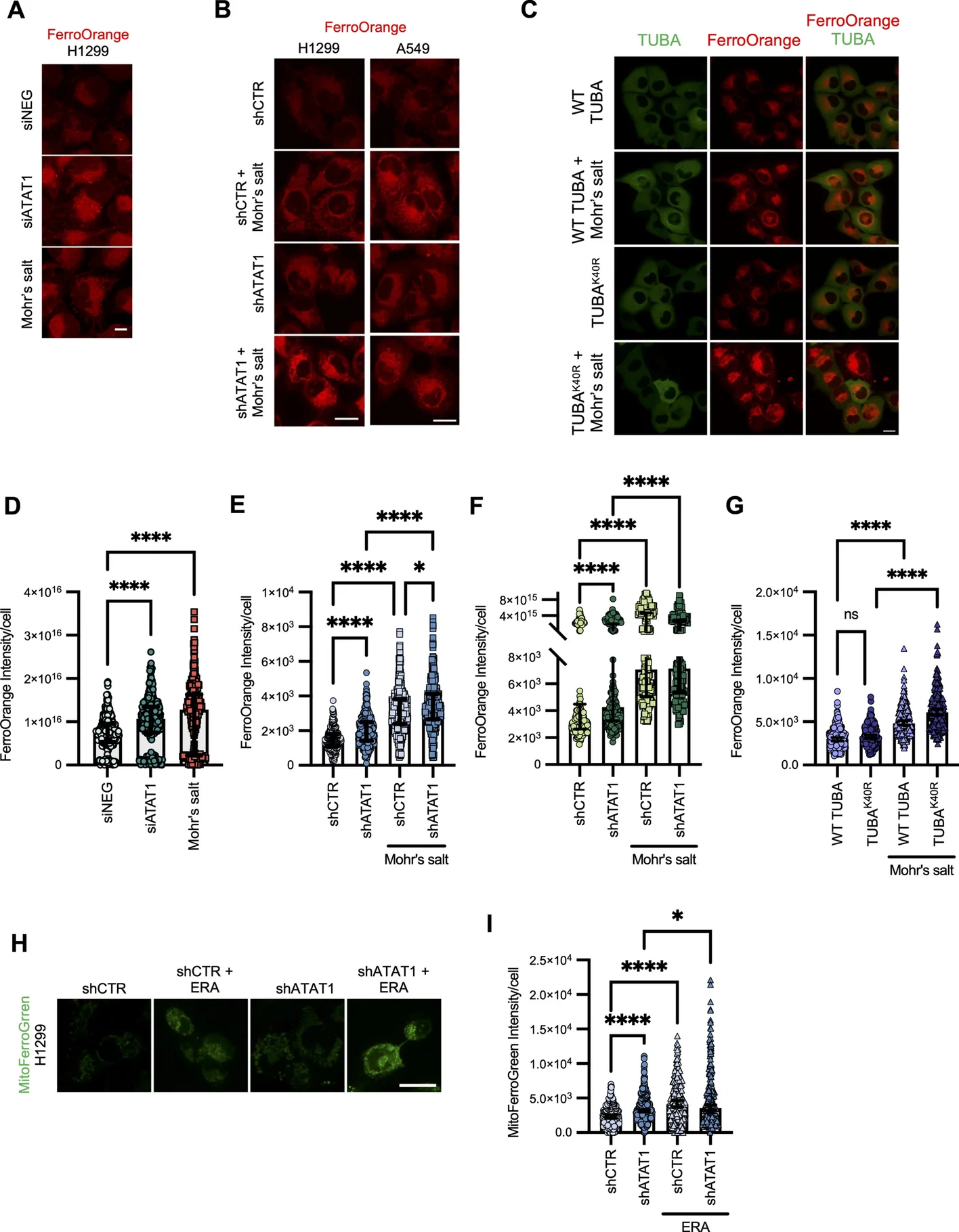
ATAT1 silencing leads to iron imbalance. **A–C** Representative images and **D–G** quantification of FerroOrange intensity/cell in the indicated cell lines and cell conditions. Ammonium iron (II) sulfate hexahydrate (Mohr’s salt) treatment for 30 min, was used as a positive control for iron accumulation. **H** Representative images and **I** quantification of MitoFerroGreen intensity/cell in the indicated cell conditions. Erastin treatment for 30 min, was used as a positive control for iron accumulation in the mitochondria. **D–G**, **I** Quantification was performed using an automated pipeline created on CellProfiler software. N > 250 cells per sample pooled from two independent experiments. Data are presented as median with interquartile range and symbols represent individual cells. Data were analyzed using a Kuskal–Wallis test followed by uncorrected Dunn’s post-hoc test. ****p < 0.0001. Scale bars: 20 µm

Autophagy activation, ROS production and mitochondrial damage, apart from mitophagy, are also implicated in ferritinophagy, a selective form of autophagy responsible for the maintenance of iron homeostasis in the cell [6]. Thus, we decided to deeply investigate this pathway in response to ATAT1 silencing. Using FerroOrange fluorescent dye to detect the overall level of cellular ferrous iron (Fe^2+^), we observed a stronger fluorescence intensity of FerroOrange dye in H1299 ATAT1-silenced cells compared with controls (Fig. 4A, D). As positive control of FerroOrange, cells were incubated with Mohr’s salts, which increases the labile Fe^2+^ ions.

**Fig. 5:**
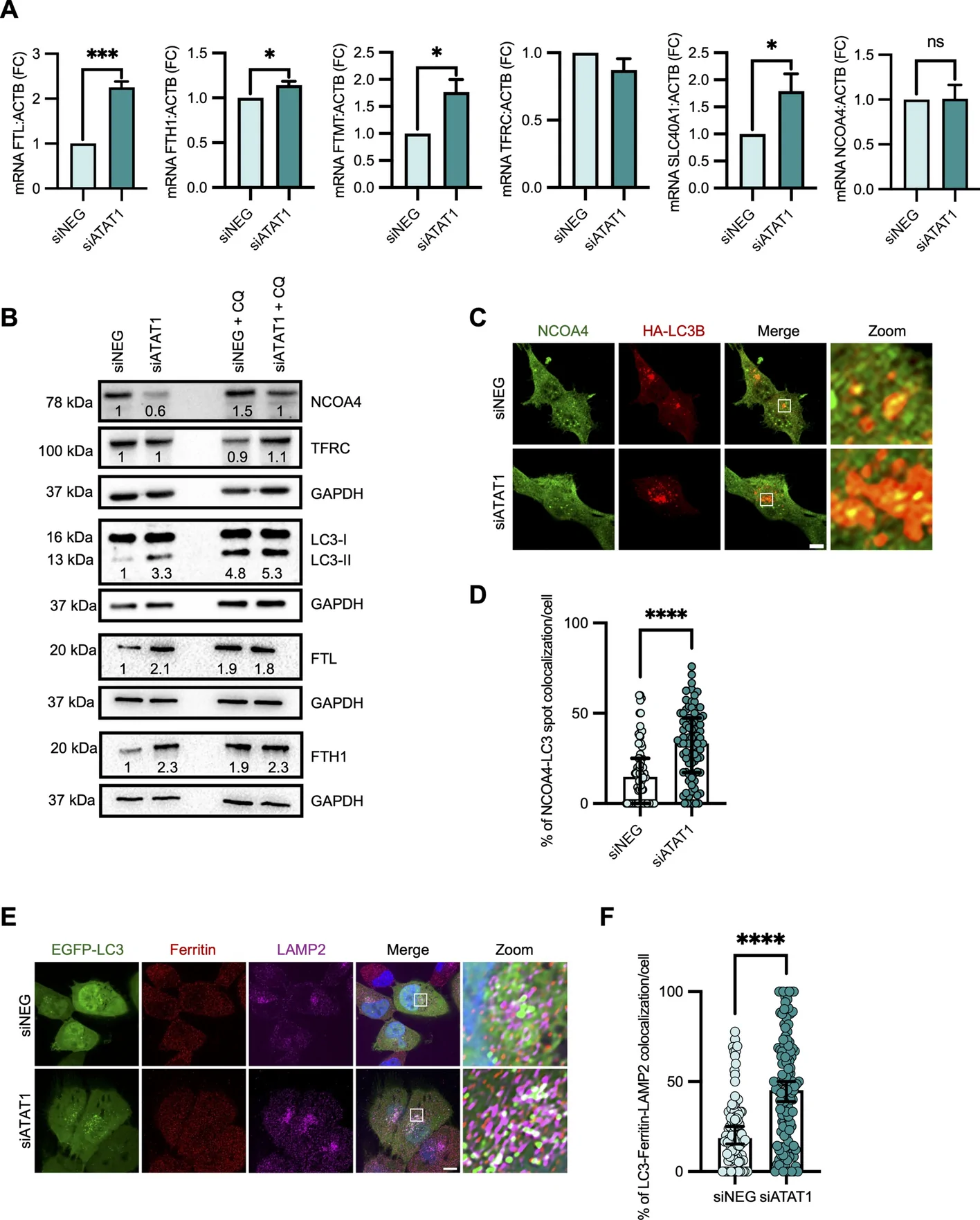
ATAT1 silencing leads to ferritinophagy activation. **A** RT-qPCR of ferritin light chain (FTL), ferritin heavy chain (FTH), mitochondrial ferritin (FTMT), transferrin receptor (TFRC) ferroportin (SLC40A1), and NCOA4 receptor mRNA in H1299 cells transfected with siNEG or siATAT1. mRNA values were normalized over ACTNB value and presented as mean ± SEM (*n* = 3). Data were analyzed using an unpaired *t* test. *p < 0.05, ***p < 0.001. **B** Immunoblot analysis of NCOA4 receptor, TFRC, FTL, FTH, and LC3-I to LC3-II conversion in H1299 cells transfected with siNEG or siATAT1 with or without 25 µM CQ for 3 h. **C** Representative NCOA4 immunofluorescence images of siNEG or siATAT1 H1299 cells expressing HA-LC3. Scale bar: 10 µm. Insets show a zoom enlargement of the boxed areas. **D** Quantification of the percentage of NCOA4 dots colocalizing with LC3 dots in cells treated as in (C). *n* > 80 cells per sample pooled from two independent experiments. **E** Representative immunofluorescence images of H1299 EGFP-LC3 cells transfected with siNEG or siATAT1 for 48 h and stained with anti-ferritin and anti-LAMP2 antibodies. Scale bar: 10 µm. Insets show a zoom enlargement of the boxed areas. **F** Quantification of the percentage of LAMP2 and ferritin dots colocalizing with EGFP-LC3 dots in cells transfected with siNEG or siATAT1. Cells were counterstained with DAPI. *n* > 80 cells per sample pooled from two independent experiments. Data are presented as median with interquartile range. **C–F** Colocalization analysis of spots was performed using the ComDet plugin of Fiji ImageJ. Data were analyzed using a Kuskal–Wallis test followed by uncorrected Dunn’s post-hoc test. *p < 0.05, **p < 0.01, ***p < 0.001, ****p < 0.0001

A similar increase was observed in both H1299 (Fig. 4B, E) and A549 (Fig. 4B, F) cells stably silenced for ATAT1 when compared with relative controls, confirming an iron overload in response to ATAT1 downregulation. Notably, no significant difference in the pool of labile iron was observed in either WT or mutated TUBA cells, supporting the results showing that key phenotypes observed in ATAT1-silenced cells are absent in cells with non-acetylatable TUBA (Fig. 4C, G). Interestingly, a marked accumulation of mitochondrial ferrous iron, stained with the MitoFerroGreen dye, was observed in ATAT1-silenced cells compared to controls (Fig. 4H, I). As positive control cells were incubated Erastin, which increases mitochondrial ferrous iron. Next, analysis of iron homeostasis genes revealed that ATAT1 silencing significantly upregulated mRNAs encoding iron storage and export proteins, including ferritin light chain (FTL) and ferritin heavy chain 1 (FTH1), ferritin mitochondrial (FTMT), and solute carrier family 40 member 1/ferroportin 1 (SLC40A1/FPN1), whereas the expression of transferrin receptor (TFRC) and of ferritin-specific autophagic receptor NCOA4 remained unchanged in siATAT1 cells (Fig. 5A).

To investigate whether the elevated ferritin and iron cell content were associated with an increased autophagic ferritin turnover [14, 21], we first examined the levels of NCOA4 and iron homeostasis proteins in basal conditions or in the presence of CQ. Western blot analysis confirmed increased FTL and FTH1 levels in ATAT1-silenced cells (Fig. 5B and Supplementary Fig. S3G for quantification**),** suggesting that the excessive amount of intracellular free iron pool may be caused by an enhanced ferritin synthesis [63]. Unexpectedly, ATAT1-silenced cells exhibited significantly reduced NCOA4 protein levels. Notably, CQ-mediated autophagy inhibition elevated NCOA4 and ferritin levels in both ATAT1-silenced and control cells, suggesting NCOA4 specific autophagic degradation in both conditions (Fig. 5B and Supplementary Fig. S3G for quantification**)**. Further, colocalization analysis between NCOA4 and LC3 puncta and between Ferritin, LC3 puncta, and lysosome associated membrane protein 2 (LAMP2) demonstrated an increased recruitment of NCOA4 on autophagosomes (Fig. 5C, D), and revealed the recruitment of ferritin to the autophagolysosome (Fig. 5E, F) following ATAT1 silencing. Collectively, these data suggest that ATAT1 silencing induces an imbalance in iron homeostasis and promotes NCOA4-mediated ferritinophagy.

### ATAT1 silencing enhances ferroptosis sensitivity

To further characterize the role of ATAT1 in iron homeostasis, we analyzed the relationship between ATAT1-dependent autophagy induction and ferroptosis in cells treated with RSL3, a well-known inducer of ferroptosis that inhibits glutathione peroxidase 4 (GPX4) [14, 64]. We investigated the effect of RSL3 on the viability of H1299 and A549 cells, treated with various concentrations of RSL3 for 24 h. We found that RSL3 reduced cell viability in H1299 cells in a dose-dependent manner and that A549 cells were more resistant to RSL3 (Supplementary Fig. S4A), as previously reported [65].

**Fig. 6:**
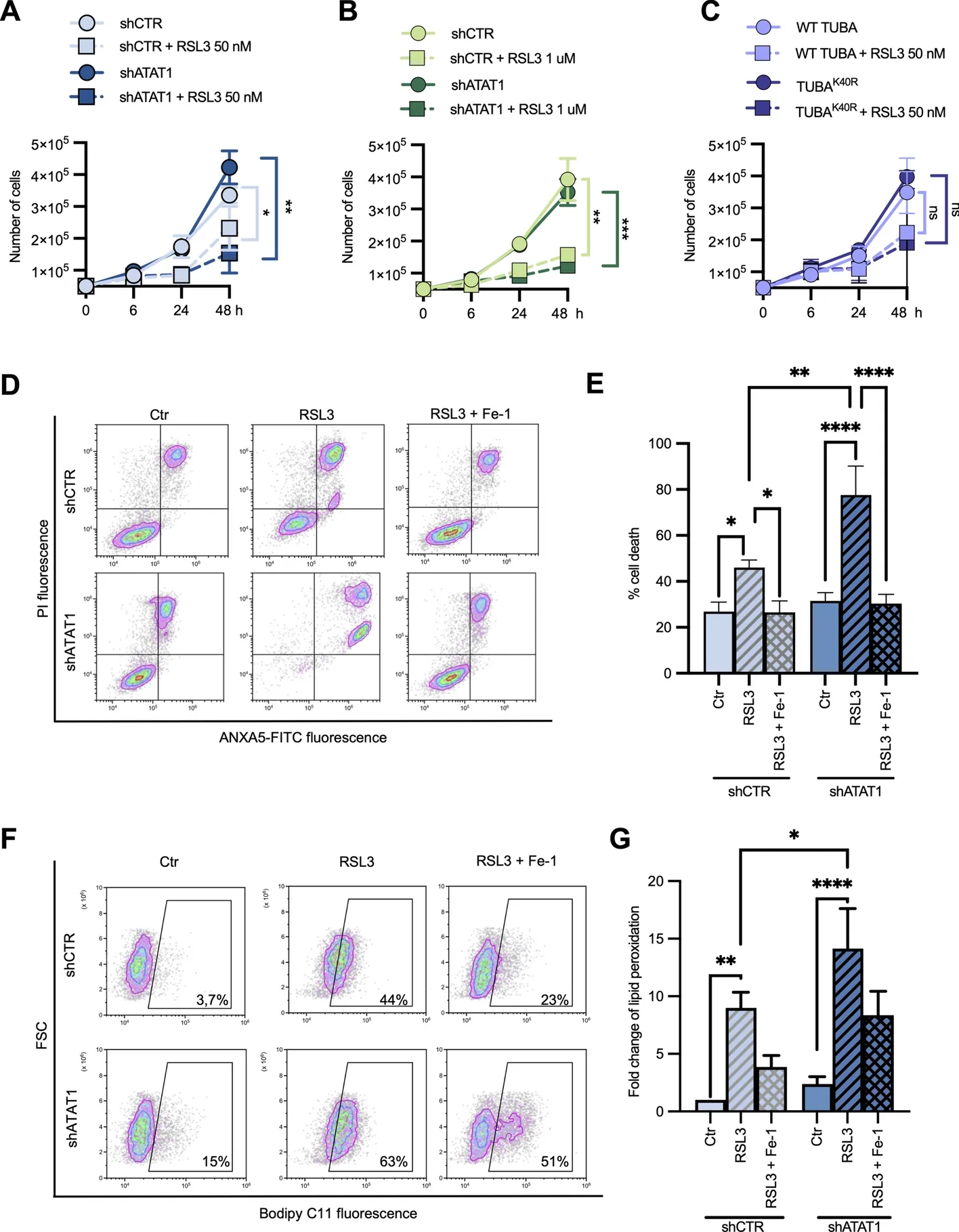
ATAT1 silenced cells are more susceptible to RSL3 treatment compared with control silenced cells. **A** Cell proliferation analysis of H1299 cells and **B** A549 cells stably transfected with shRNA targeting ATAT1 (shATAT1) or with scramble shRNA (shCTR) alone or in combination with 50 nM RSL3 (H1299 cells) or 1 µM RSL3 (A549 cells). Data are presented as mean ± SEM from at least two independent experiments with triplicated counts. **C** Cell proliferation analysis of H1299 cells expressing WT TUBA or the mutant form TUBA^K40R^ with or without 50 nM RSL3. Data are presented as mean ± SEM from three independent experiments with triplicated counts. **A–C** The area under the curve (AUC) parameter was used to analyze the data using an ordinary one-way ANOVA followed by Fisher’s LSD post-hoc test. *p < 0.05, **p < 0.01, ***p < 0.001,. ns = nonsignificant. **D** Representative density plots and **E** quantification of the percentage of ANXA5-FITC positive H1299 cells stably transfected with shRNA targeting ATAT1 (shATAT1) or with a scramble shRNA (shCTR) alone or in combination with 50 nM RSL3, 1 µM Ferrostatin-1 (Fe-1), or with a combination of the two drugs. Data are presented as mean ± SEM from three independent experiments and were analyzed using an ordinary one-way ANOVA test followed by uncorrected Fisher’s LSD post-hoc test. *p < 0.05. **p < 0.01, ****p < 0.0001. **F** Density plots and **G** quantification of the percentage of oxidized Bodipy C11 positive cells, in cells treated as in (**E**). Data are presented as Fold Change ± SEM from three independent experiments and were analyzed using a non parametric Kruskal–Wallis test followed by a post-hoc uncorrected Dunn’s test. *p < 0.05, **p < 0.01, ****p < 0.0001

Then, the response of shCTR and shATAT1 H1299 cells to RSL3 treatment was analyzed in terms of cell proliferation. As assessed by growth curves analysis, no significant differences in cell proliferation were observed between shCTR and shATAT1 cells. Exposure to 50 nM RSL3 significantly reduced cell proliferation in both control and ATAT1-silenced cells in a time-dependent manner. However, ATAT1-silenced cells were more sensitive to RSL3 (Fig. 6A). Similar results were obtained in A549 cells treated with 1 µM RSL3 (Fig. 6B). Consistent with previous data on oxidative stress and autophagy, the cell growth inhibition in response to RSL3 was minimal and similar in H1299 TUBA^K40R^ cells and in H1299 WT TUBA cells (Fig. 6C), thus confirming a direct role of ATAT1 downregulation in the induction of a ferroptosis vulnerability phenotype. Remarkably, ATAT1- silencing also enhances cellular sensitivity to Erastin, another ferroptosis inducer, which inhibits system Xc^−^ activity (Supplementary Fig. S4B).

To further corroborate these results, we assessed ferroptotic cell death by annexin-V staining in shCTR and shATAT1 H1299 cells under various conditions: untreated, treated with RSL3 alone or in combination with the ferroptosis inhibitor Ferrostatin-1 (Fer-1) [14] (Fig. 6D, E). While basal cell death levels were comparable between shCTR and shATAT1 cells, RSL3 treatment induced significantly higher cell death in shATAT1 cells compared with controls. Importantly, cotreatment with Fer-1 effectively rescued RSL3-induced cell death in both control and shATAT1 cells, confirming that ferroptosis was the activated pathway. As lipid peroxidation is a hallmark of ferroptosis [66, 67], we also assessed polyunsaturated membrane fatty acids oxidation. RSL3 induced a significant increase in lipid peroxidation in both cell conditions, with shATAT1 cells displaying an increased sensitivity to RSL3-induced oxidation and lipid peroxidation was significantly rescued by Fer-1 (Fig. 6F, G). Similarly, a significant induction of cell death and lipid peroxidation by RSL3 was observed in A549 cells, with a higher sensitivity of shATAT1 cells, thus confirming that ATAT1 silencing renders cancer cells vulnerable to RSL3 (Supplementary Fig. S4C,D). Conversely, the induction of the sub-G1peak, as an index of cell death, in response to RSL3 was similar in H1299 TUBA^K40R^ cells and in H1299 WT-TUBA cells (Supplementary Fig. S4E), confirming that ATAT1 but not tubulin acetylation is involved in cell responses to ferroptosis.

**Fig. 7:**
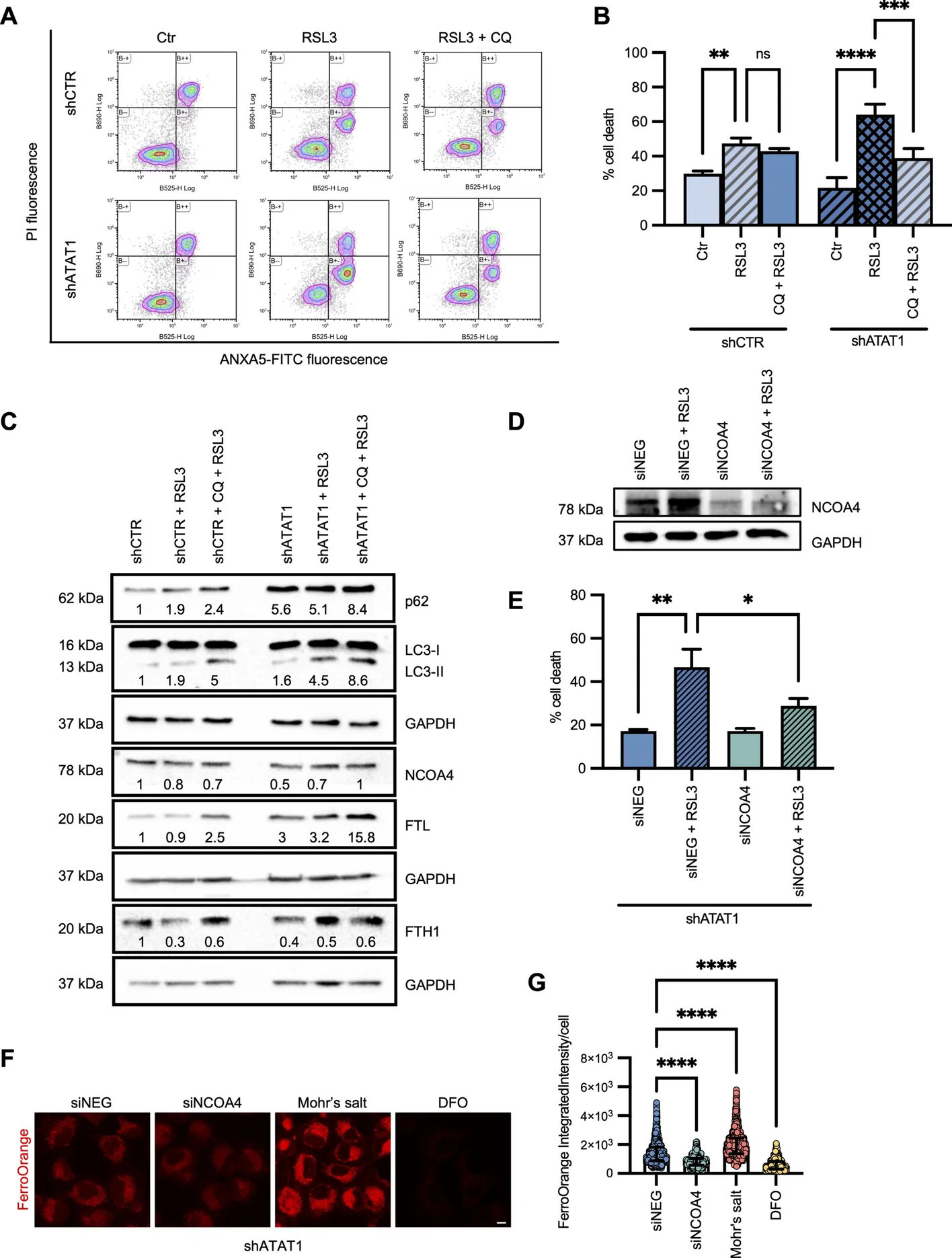
ATAT1 silenced cells are more susceptible to RSL3 due to NCOA4-mediated ferritinophagy activation. **A** Representative density plots and **B** quantification of the percentage of ANXA5 positive H1299 cells stably transfected with shRNA targeting ATAT1 (shATAT1) or with scramble shRNA (shCTR) treated with 50 nM RSL3 or with a combination of RSL3 and 10 µM CQ. Data are presented as mean ± SEM (n=3) and were analyzed using an ordinary One-Way ANOVA test followed by uncorrected Fisher’s LSD post-hoc test. *P <0.05, **P < 0.01, ****P < 0.0001. **C** Immunoblot analysis of LC3 conversion, SQSTM1/p62, NCOA4, FTL and FTH1 protein levels in shATAT1or shCTR H1299 cells treated with 50 nM RSL3, or with a combination of RSL3 and 10 µM CQ. **D** Representative immunoblot analysis of NCOA4 protein levels in shATAT1 H1299 cells transfected with a control siRNA (siNEG), a siRNA against NCOA4 (siNCOA4) for 72 h, with or without 50 nM RSL3 for the last 24 h. **E** Quantification of the percentage of ANXA5 positive H1299 shATAT1 cells, transfected as in (D). Data are presented as mean ± SEM from two independent experiments and were analyzed using an ordinary One-Way ANOVA test followed by uncorrected Fisher’s LSD post-hoc test. *P <0.05, **P < 0.01. **F** Representative images and **G** quantification of FerroOrange intensity per cell in H1299 cells stably transfected with shRNA targeting ATAT1 (shATAT1) transfected with siNEG or siNCOA4 for 72 h or treated with Ammonium iron (II) sulfate hexahydrate (Mohr’s salt) for 30 min or Deferoxamine (DFO), an iron chelator which indues ferritinophagy, for 24 h. Quantification was performed using an automated pipeline created on CellProfiler software. N ≥ 300 cells per sample pooled from two independent experiments. Scale bar: 10 µm. Data are presented as median with interquartile range and symbols represent individual cells. Data were analyzed using a Kuskal-Wallis test followed by uncorrected Dunn’s post-hoc test. ****P < 0.0001

Next, to investigate the interplay between autophagy and RSL3-induced ferroptosis, we assessed whether autophagic flux inhibition affected RSL3-induced cell death. Interestingly, RSL3-induced cell death was rescued by the presence of CQ in ATAT1-silenced cells (Fig. 7A, B). At the same time, RSL3 treatment increased LC3-II and SQSTM1/p62 levels in shCTR cells, confirming autophagy modulation by RSL3 (Fig. 7C). Moreover, RSL3 increased both LC3-II and SQSTM1/p62 levels in shATAT1 cells beyond those observed in shCTR cells (Fig. 7C). Notably, treatment with CQ in combination with RSL3 further elevated the expression of autophagic markers and ferritinophagy-related proteins in shATAT1 cells compared with shCTR cells, consistent with their enhanced sensitivity to RSL3 (Fig. 7C and Fig. S5A for quantification).

Finally, to confirm the role of NCOA4-mediated ferritinophagy in enhancing the sensitivity of ATAT1-silenced cells to ferroptosis, we analyzed cell death and iron accumulation in response to NCOA4 silencing in ATAT1-downregulated cells. Consistent with our previous data, NCOA4 silencing significantly reduced cell death in ATAT1 stably silenced cells (Fig. 7D, E). Concordantly, NCOA4 silencing reduced iron accumulation in ATAT1-silenced cells (Fig. 7F, G). Taken together, these findings demonstrate that ATAT1 silencing increases cellular sensitivity to ferroptosis induction by increasing NCOA4-mediated ferritinophagy.

## Discussion

In this study, we demonstrated that ATAT1, the enzyme responsible for K40 α-tubulin acetylation, plays a previously unrecognized role in the regulation of ferritinophagy, revealing its importance in iron homeostasis. Moreover, we also demonstrated that ATAT1-silenced cells exhibit increased sensitivity to RSL3, a well-established ferroptosis inducer, a finding with potential therapeutic implications.

Several reports have highlighted the role of K40 acetylation in response to various stresses, including oxidative stress, drugs, and nutrient starvation [43, 68–71]. According to these studies, different insults can alter the levels or activity of tubulin deacetylases or ATAT1 to promote the rapid acetylation of microtubules, as a key cell adaptation to stress. Earlier findings demonstrated that ROS/AMPK activation can enhance ATAT1 activity and microtubule acetylation [69]. However, it is unclear how cells respond to the absence of ATAT1. Here, we found that ATAT1 silencing is associated with increased ROS levels, which activates AMPK signaling and subsequently increases autophagic flux, suggesting a potential regulative loop. Indeed, treatment with a ROS scavenger reduced AMPK activation, LC3 lipidation, and the autophagic receptor SQSTM1/p62 protein levels in ATAT1-silenced cells, indicating that autophagy activation in ATAT1-silenced cells is owing to elevated basal ROS levels. Unexpectedly, we found a transcriptional activation of SQSTM1/p62 [59], indicating that ATAT1 regulates SQSTM1/p62 levels independently of autophagy. This observation is in line with other reports showing an upregulation of p62/SQSTM1 mRNA and protein in response to oxidative and ER stress stimuli [72–74].

Our study also shows, for the first time, that ATAT1 silencing affects cellular bioenergetics, as evidenced by reduced ATP production, decreased oxygen consumption, and substantial alterations in mitochondrial dynamics. These observations suggest that ATAT1 may function as a key regulator balancing redox homeostasis. We also demonstrated an unrecognized role for ATAT1 in iron metabolism, providing evidence for ATAT1-dependent regulation of genes that control iron trafficking and storage. Indeed, ATAT1-silenced cells exhibited iron overload, increased levels of ferritin and SLC40A1/FPN, indicating that the excessive amount of intracellular free iron in siATAT1 cells results in an enhanced ferritin synthesis, in order to store more iron and protect cells from oxidative damage [63]. On the other hand, we also observed a mitochondrial iron overload which may cause the elevated ROS levels found in ATAT1-silenced cells through the Fenton reaction, leading to oxidative stress and, in turn, activating ATG7‐dependent selective autophagy to restore cellular iron homeostasis by NCOA4 degradation. While our data links ATAT1-deficiency to mitochondrial dysfunction and autophagy via modulation of ROS and iron metabolism, it cannot be ruled out that ATAT1 may exert further effects on the autophagy pathway, e.g. by acetylating or interacting with proteins of the autophagic machinery or acting as a canonical acetyltransferase to modulate the transcription of antioxidant or metabolic genes. The increased production of ROS, together with the excess of iron in the cell, might render cancer cells more vulnerable to ferroptosis. Accordingly, we were able to demonstrate that ATAT1-silenced cells increased lipid peroxidation and mitochondrial dysfunction and are more sensitive to the treatment with ferroptosis inducers. Additionally, both pharmacological blockage of autophagy and NCOA4 silencing significantly reduced RSL3-induced cell death in ATAT1-silenced cells, confirming that ATAT1 silencing confers ferroptosis susceptibility in different cell types through the activation of NCOA4-mediated ferritinophagy [6, 12, 75].

Finally, experiments using cells expressing non-acetylatable TUBA provided important mechanistic insights by demonstrating that the key phenotypes observed following ATAT1 silencing are not recapitulated in cells lacking K40 acetylation. Indeed, cells expressing wild type or mutated TUBA showed similar accumulation of LC3-II under basal conditions, thus suggesting that the loss of K40 acetylation of TUBA is not involved in the autophagy phenotypes observed in the absence of ATAT1. No significant differences were also observed in terms of iron accumulation and RSL3 susceptibility in K40 mutated cells compared with WT-TUBA cells. These findings indicate that ATAT1 deficiency acts in modulating ROS production, activating NCOA4-mediated ferritinophagy and enhancing ferroptosis sensitivity independently of its tubulin acetylation activity.

Altogether, our data identify ATAT1 as a previously unrecognized regulator within the ferritinophagy-dependent ferroptosis pathway. However, the mechanistic role of ATAT1 in this pathway is still unclear. Further investigation will be essential to: i) clarify the possible ATAT1 targets in this pathway; ii) validate ATAT1 inhibition as a therapeutic strategy in cancer; iii) support the future development of selective ATAT1 inhibitors, which have not been identified so far.

## Conclusions

In this study, we investigated the role of ATAT1 in oxidative stress and autophagy. Our results demonstrated that ATAT1 silencing increases the formation of LC3-positive autophagosomes and modulates the expression of several autophagic markers. Interestingly, ATAT1-silenced cells also produce a significantly higher amount of ROS and lipid peroxides, accompanied by alterations in mitochondrial dynamics, under both basal and stress conditions. Mechanistically, ATAT1 silencing alters iron homeostasis and induces ferritinophagy. In line with these findings, ATAT1 silencing sensitizes lung cancer cells to ferroptotic cell death.

Overall, this study provides new insights into the role of ATAT1 in cancer cells, broadening its recognized role in microtubule acetylation to encompass significant contributions to autophagy, ferroptosis and iron homeostasis.

## Supplementary Information

Supplementary Material 1

Supplementary Material 2

Supplementary Material 3

Supplementary Material 4

Supplementary Material 5

Supplementary Material 6

Supplementary Material 7

Supplementary Material 8

Supplementary Material 9

## Data Availability

The datasets used and/or analyzed during the current study are available from the corresponding author on reasonable request.

## Abbreviations

AMPK: AMP-activated protein kinase
ATAT1: α-Tubulin acetyltransferase 1
CQ: Chloroquine
ECAR: Extracellular acidification rate
FCCP: Carbonyl cyanide 4-(trifluoromethoxy) phenylhydrazone
Fer-1: Ferrostatin-1
FTH1: Ferritin heavy chain 1
FTL: Ferritin light chain
FTMT: Ferritin mitochondrial
H_2_O_2_: Hydrogen peroxide
HBSS: Hank’s Buffer Salt Solution
LC3: Microtubule-associated protein 1 light chain 3
MMP: Mitochondrial membrane potential
Mohr’s salt: Ammonium iron(II) sulfate hexahydrate
MTs: Microtubules
NAC: N-acetyl-l-cysteine
NCOA4: Nuclear receptor coactivator 4
NH_4_Cl: Ammonium chloride
OCR: Oxygen consumption rate
OXPHOS: Oxidative phosphorylation
PRKN: Parkin RBR E3 ubiquitin protein ligase
ROS: Reactive oxygen species
SLC40A1/FPN1: Solute carrier family 40 member 1/ferroportin 1
SQSTM1/p62: Sequestosome 1
TFRC: Transferrin receptor
TOMM20: Translocase of outer mitochondrial membrane 20
TUBA: α-Tubulin
