# Supplementary material for: α-tubulin N-acetyltransferase 1 regulates NCOA4-mediated ferritinophagy and protects cancer cells from ferroptosis
Source: Cell Mol Biol Lett. 2026 Jun 12;31:134. doi: 10.1186/s11658-026-00964-2 (PMC13520228; doi:10.1186/s11658-026-00964-2)

Figure 1A – H1299

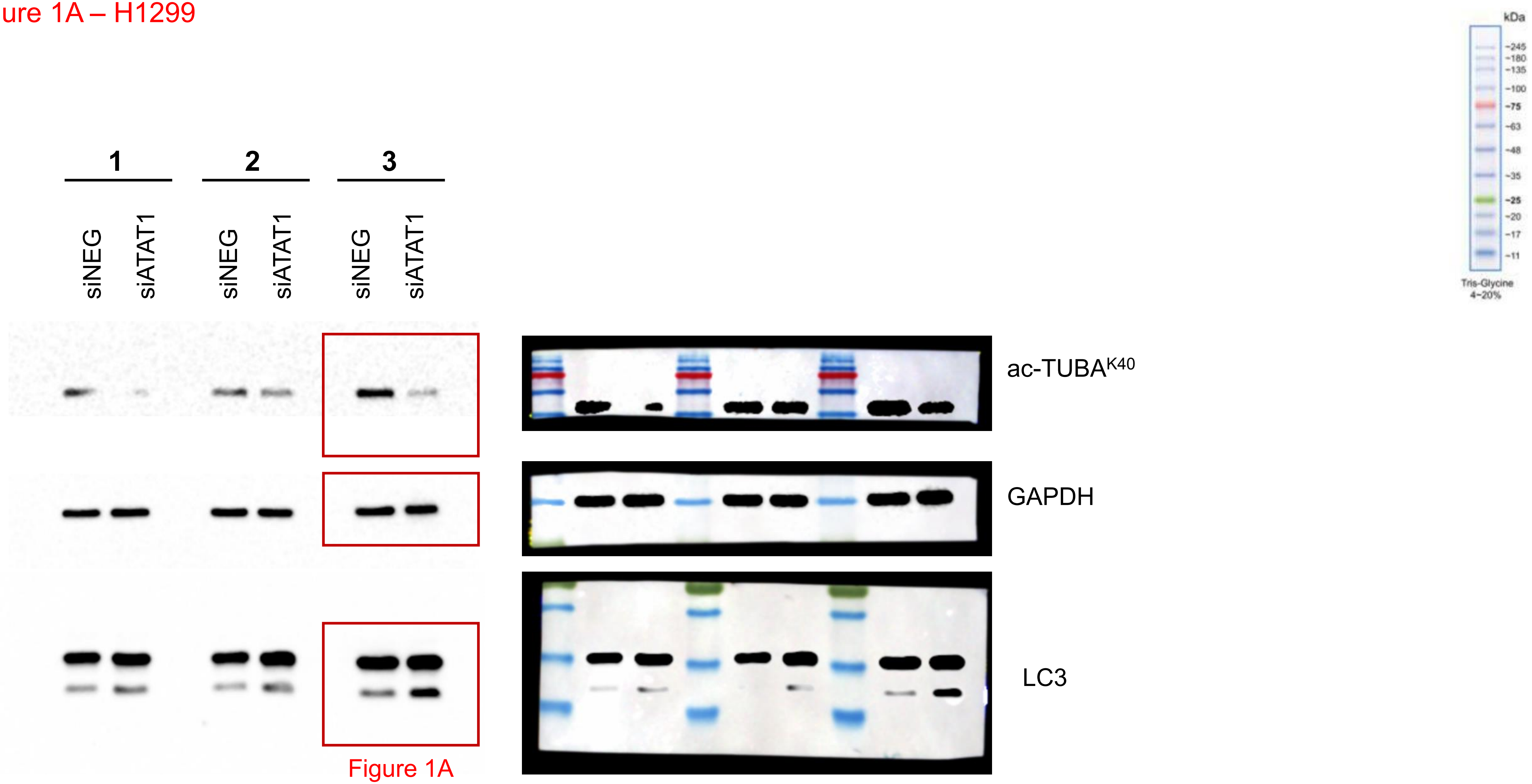

Figure 1A - A549

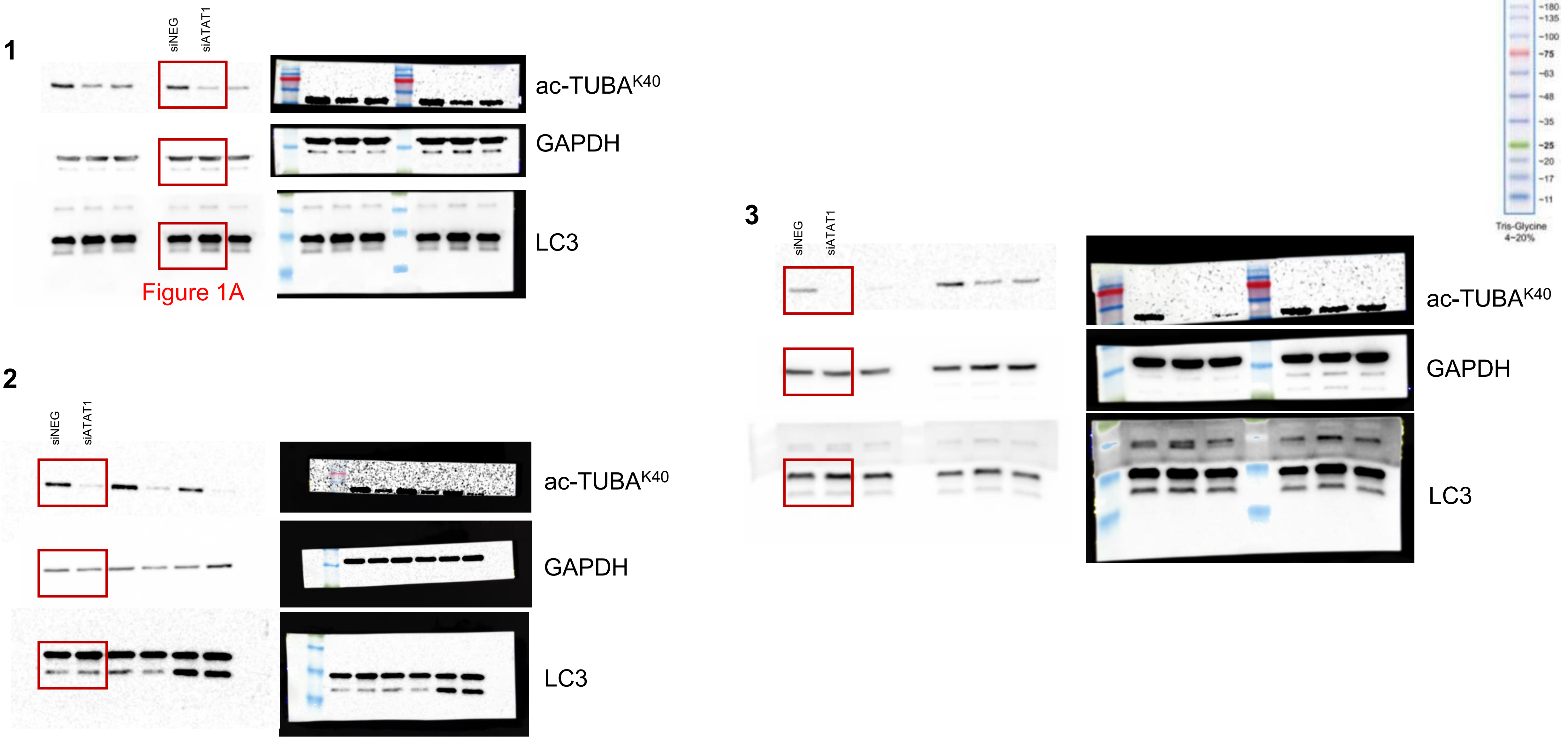

Figure 1A - HeLa

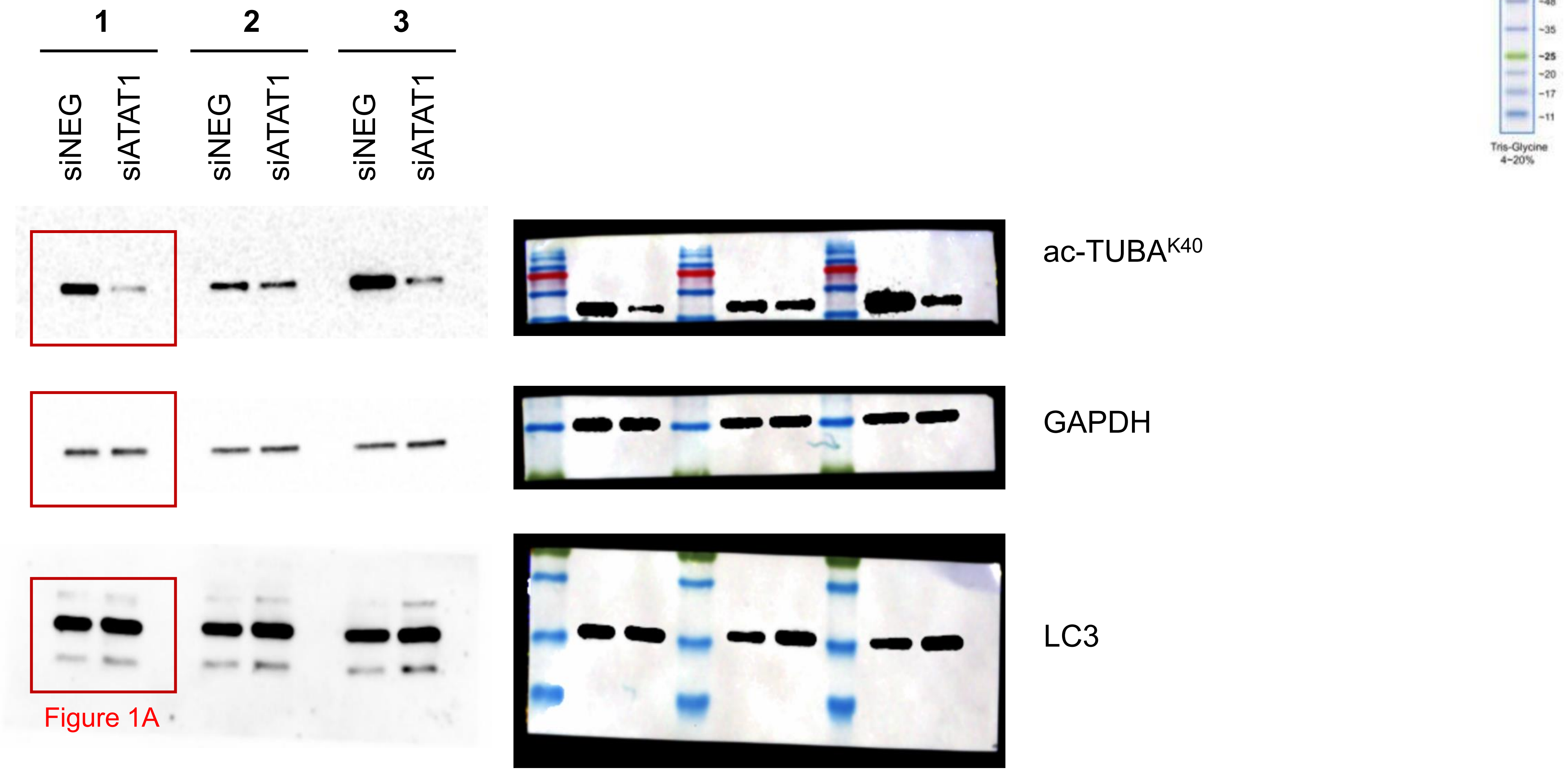

Figure 1H

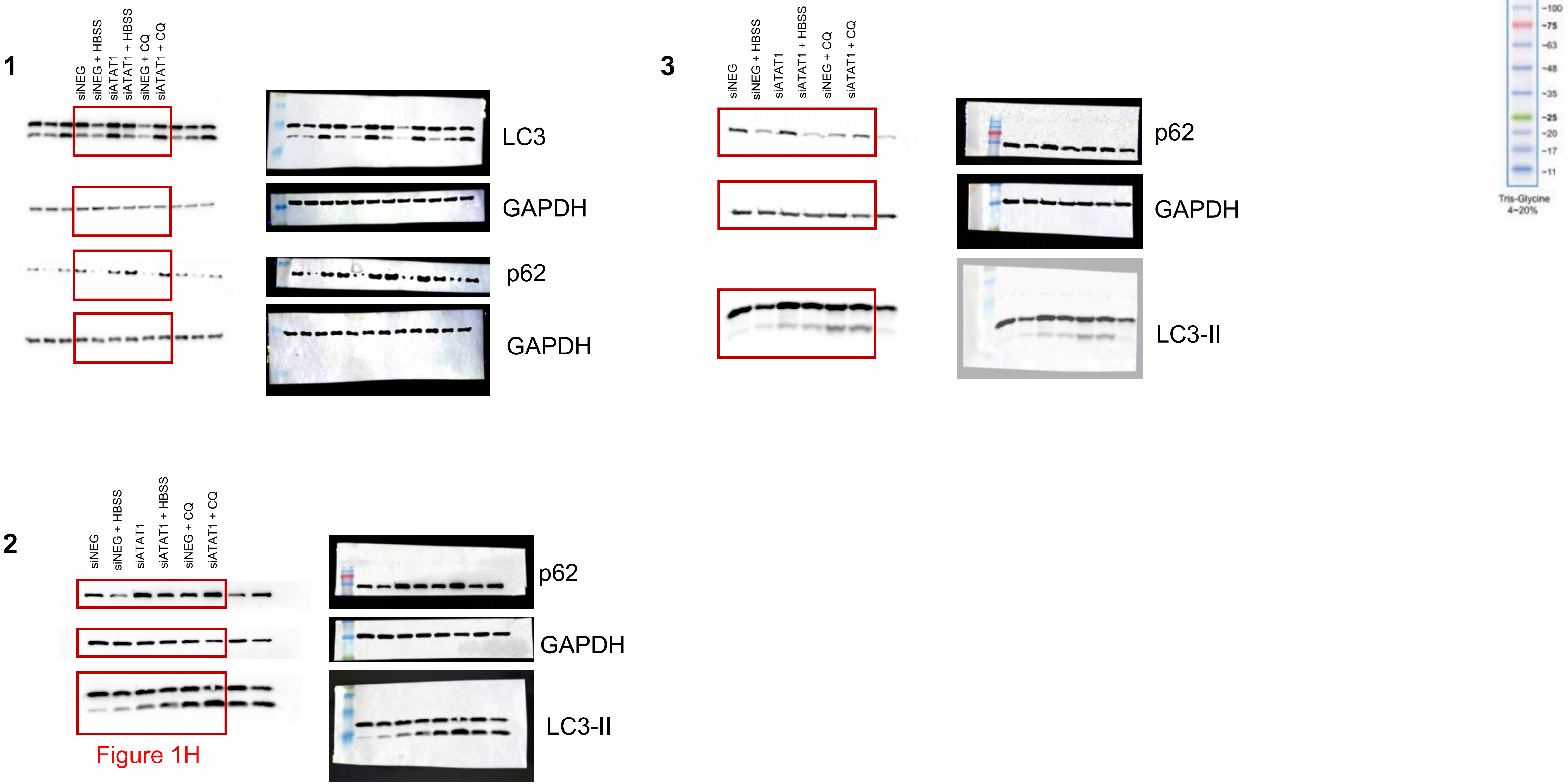
1
siNEG
siNEG + HBSS
siATAT1
siATAT1 + HBSS
siNEG + CQ
siATAT1 + CQ
LC3
GAPDH
p62
GAPDH
3
siNEG
siNEG + HBSS
siATAT1
siATAT1 + HBSS
siNEG + CQ
siATAT1 + CQ
p62
GAPDH
LC3-II
kDa
245
180
135
100
75
63
48
35
25
20
17
11
Tris-Glycine
4~20%
2
siNEG
siNEG + HBSS
siATAT1
siATAT1 + HBSS
siNEG + CQ
siATAT1 + CQ
p62
GAPDH
LC3-II
Figure 1H

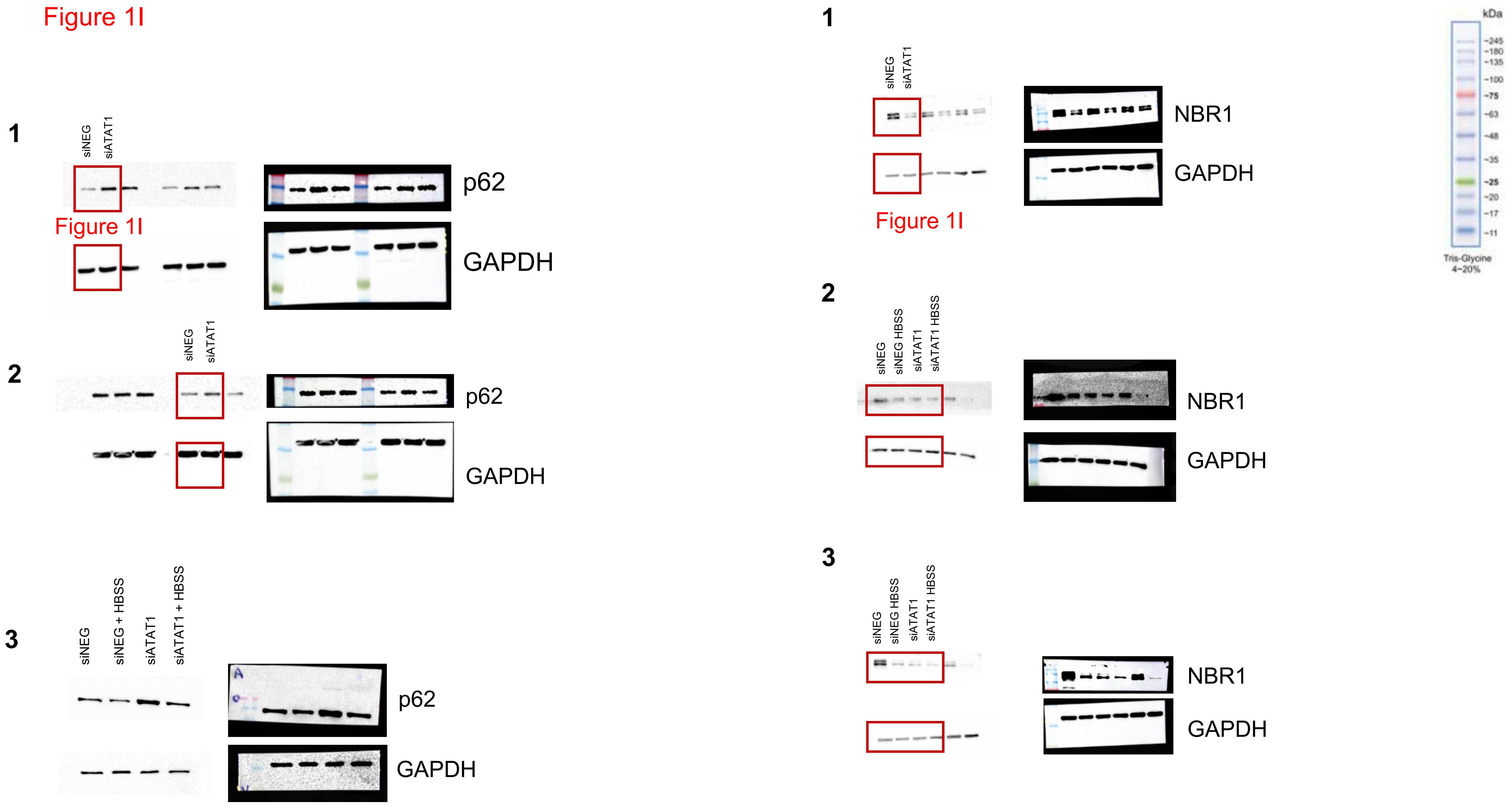
Figure 1I
1
siNEG
siATAT1
p62
Figure 1I
GAPDH
2
siNEG
siATAT1
p62
GAPDH
3
siNEG
siNEG + HBSS
siATAT1
siATAT1 + HBSS
p62
GAPDH
1
siNEG
siATAT1
NBR1
GAPDH
Figure 1I
2
siNEG
siNEG HBSS
siATAT1
siATAT1 HBSS
NBR1
GAPDH
3
siNEG
siNEG HBSS
siATAT1
siATAT1 HBSS
NBR1
GAPDH
kDa
245
180
135
100
75
63
48
35
25
20
17
11
Tris-Glycine
4~20%

Figure 1L

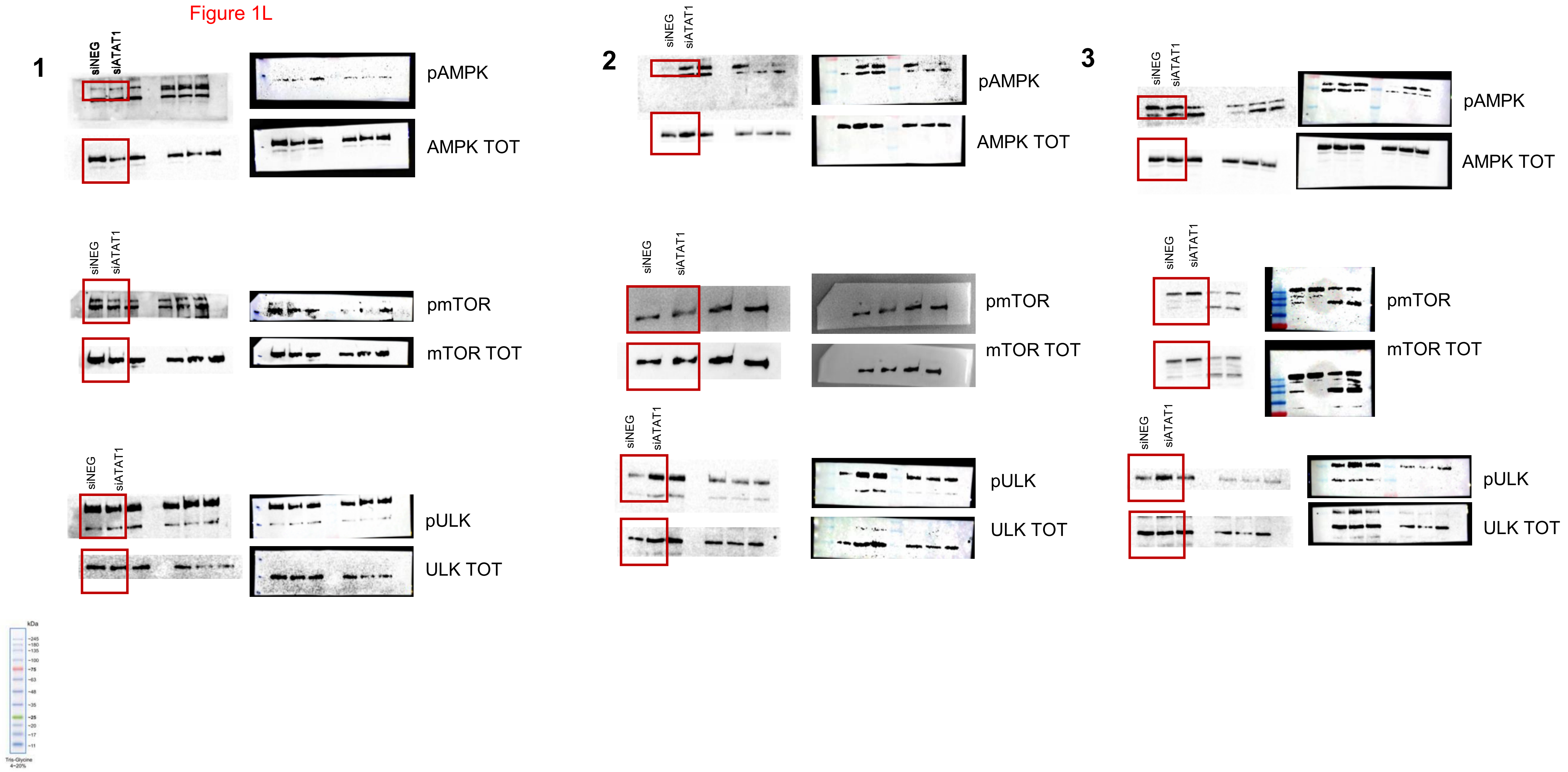

Figure 1L
1
siNEG
siATAT1
pAMPK
AMPK TOT
pmTOR
mTOR TOT
pULK
ULK TOT
2
siNEG
siATAT1
pAMPK
AMPK TOT
pmTOR
mTOR TOT
pULK
ULK TOT
3
siNEG
siATAT1
pAMPK
AMPK TOT
pmTOR
mTOR TOT
pULK
ULK TOT

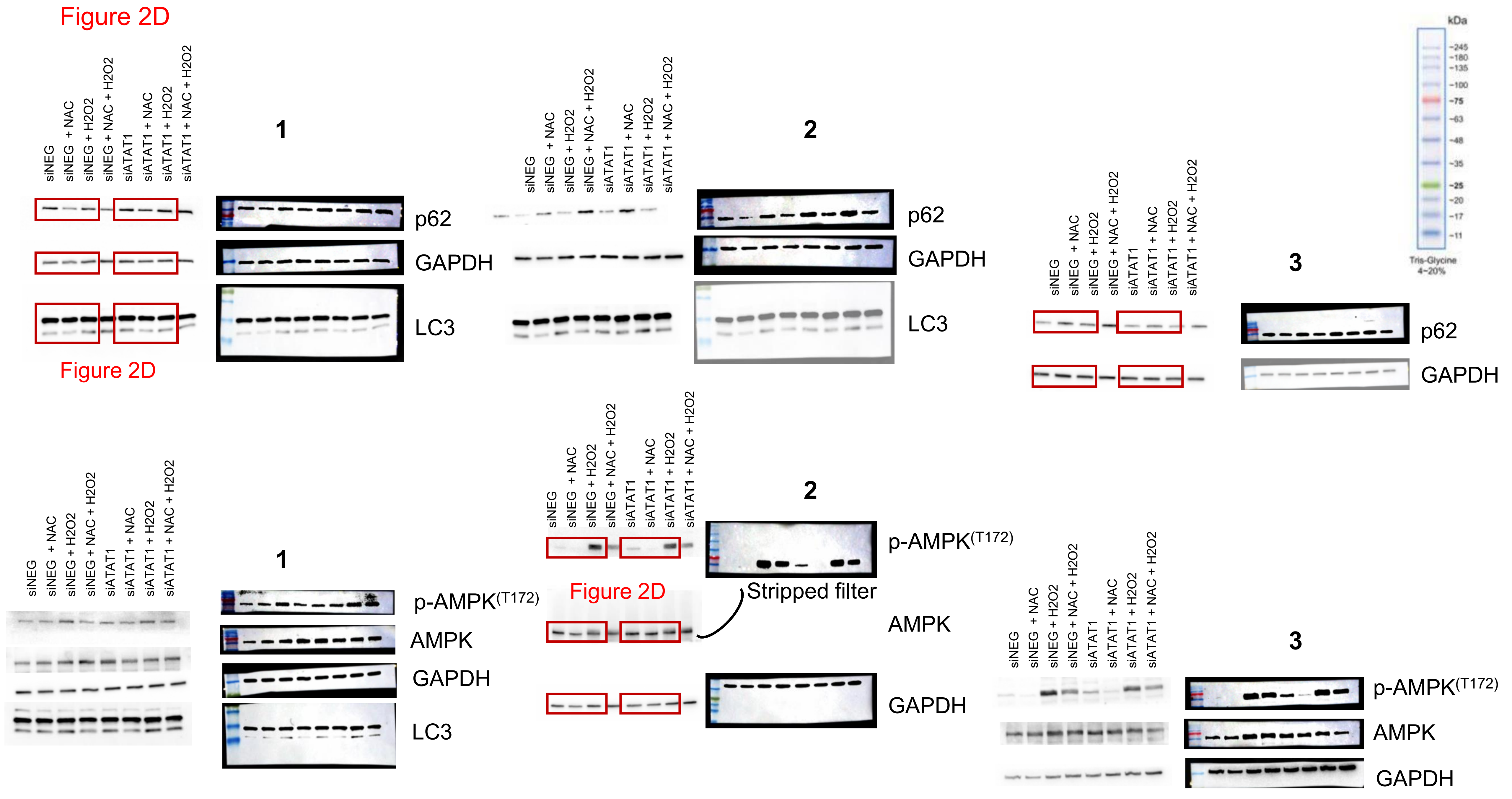

Figure 2D
siNEG
siNEG + NAC
siNEG + H2O2
siNEG + NAC + H2O2
siATAT1
siATAT1 + NAC
siATAT1 + H2O2
siATAT1 + NAC + H2O2
1
2
3
p62
GAPDH
LC3
Figure 2D
kDa
245
180
135
100
75
63
48
35
25
20
17
11
Tris-Glycine 4~20%
p-AMPK(T172)
AMPK
Figure 2D
Stripped filter

Figure 2E

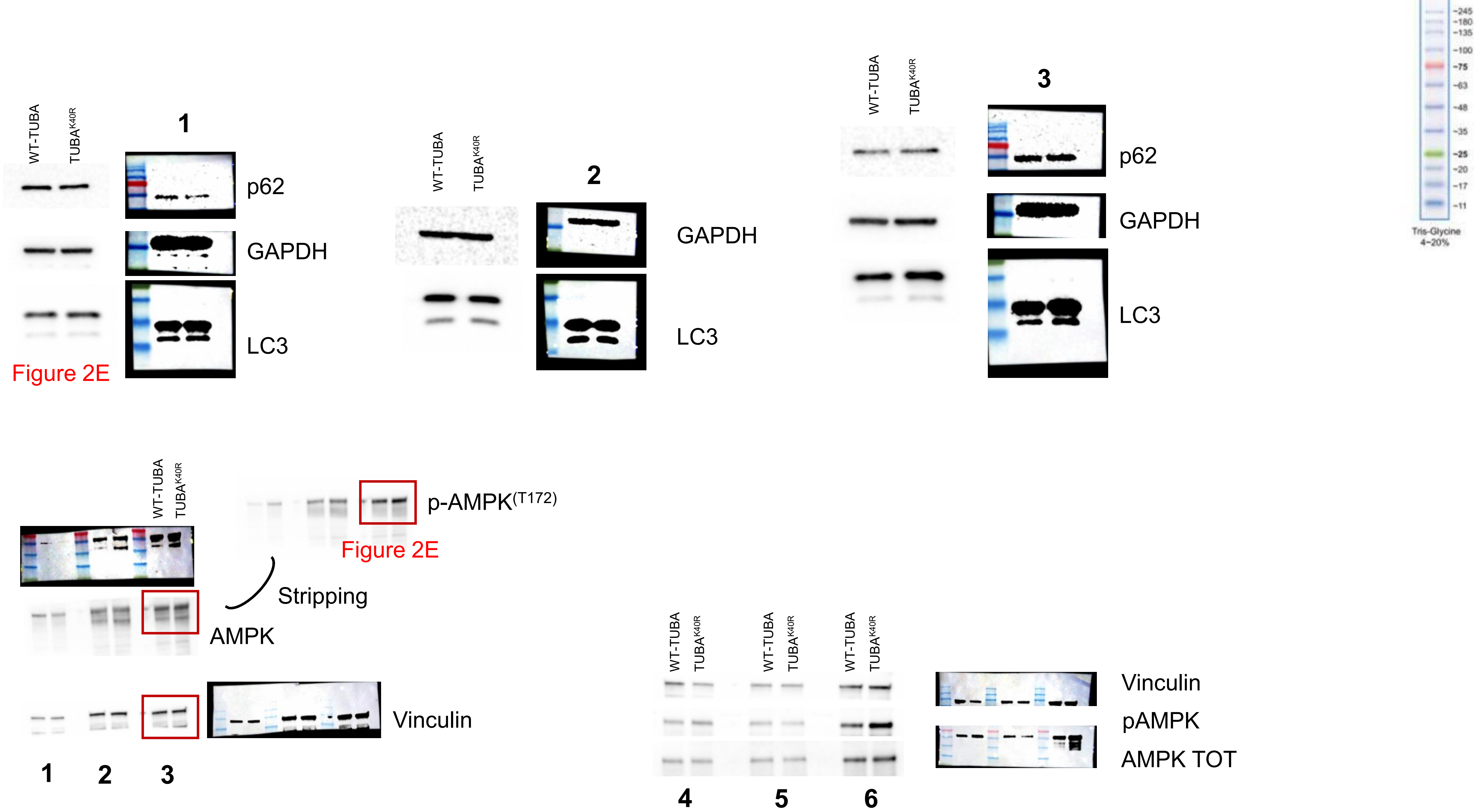

1
2
3
WT-TUBA
TUBA^{K40R}
p62
GAPDH
LC3
Figure 2E
kDa
245
180
135
100
75
63
48
35
25
20
17
11
Tris-Glycine 4~20%
p-AMPK$^{(T172)}$
Figure 2E
Stripping
AMPK
Vinculin
1
2
3
4
5
6
Vinculin
pAMPK
AMPK TOT

Figure 5B

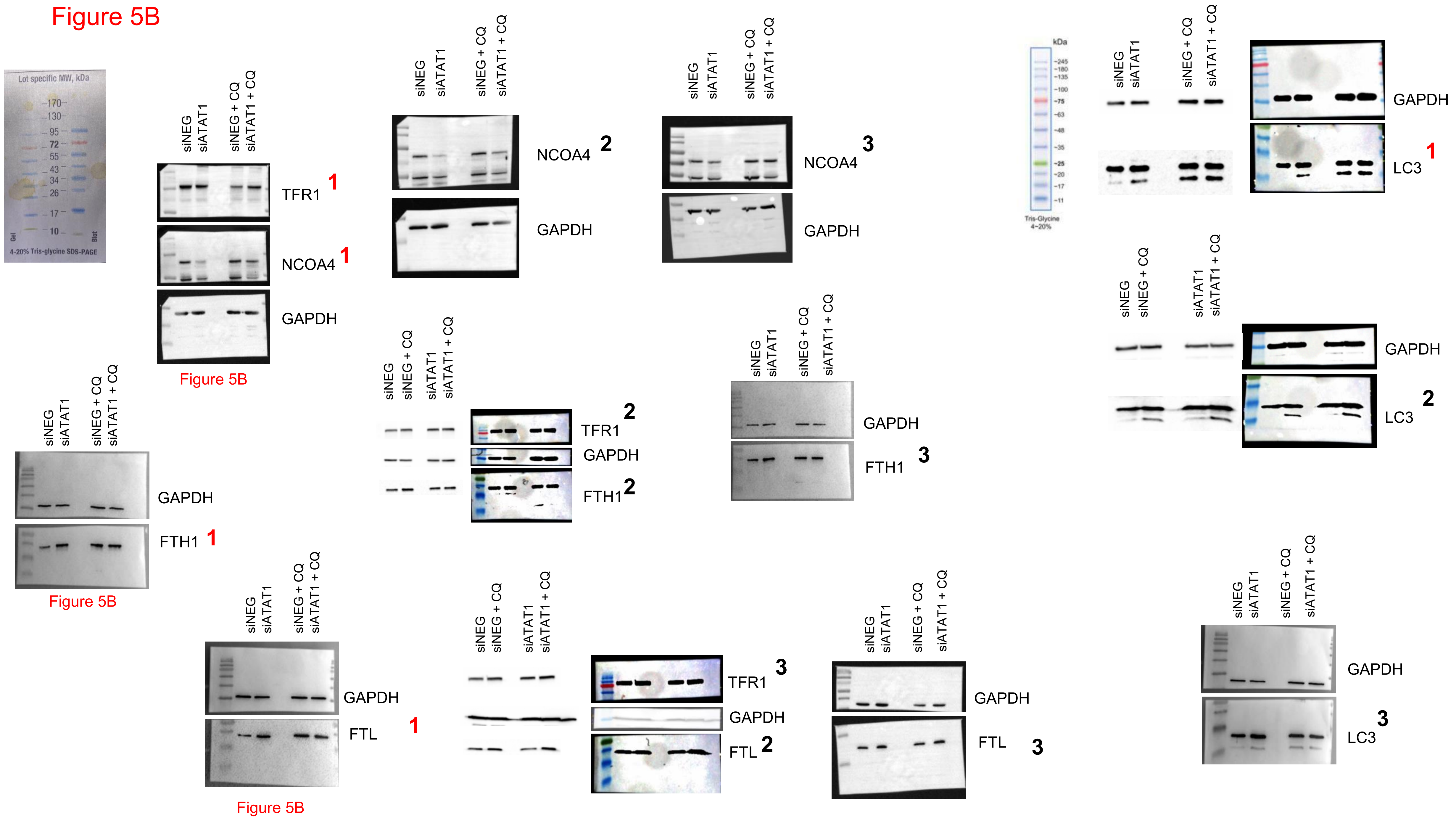

Lot specific MW, kDa
170
130
95
72
55
43
34
26
17
10
Gel
Blot
4-20% Tris-glycine SDS-PAGE
siNEG
siATAT1
siNEG + CQ
siATAT1 + CQ
TFR1 1
NCOA4 1
GAPDH
Figure 5B
NCOA4 2
GAPDH
NCOA4 3
GAPDH
kDa
245
180
135
100
75
63
48
35
25
20
17
11
Tris-Glycine 4~20%
GAPDH
LC3 1
GAPDH
LC3 2
GAPDH
FTH1 1
Figure 5B
TFR1 2
GAPDH
FTH1 2
GAPDH
FTH1 3
GAPDH
FTL 1
Figure 5B
TFR1 3
GAPDH
FTL 2
GAPDH
FTL 3
GAPDH
LC3 3

Figure 7C p62/LC3

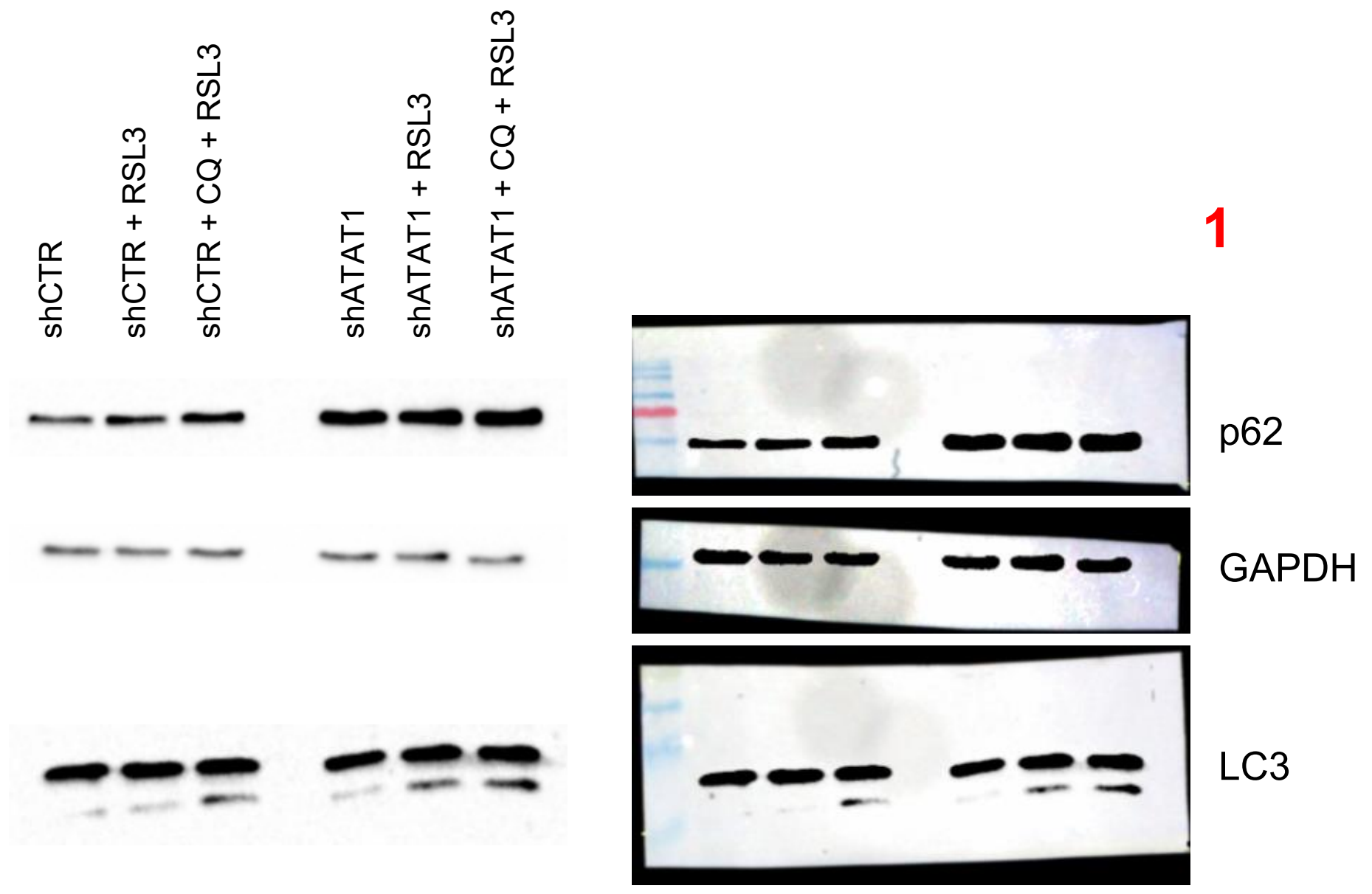

Figure 7C

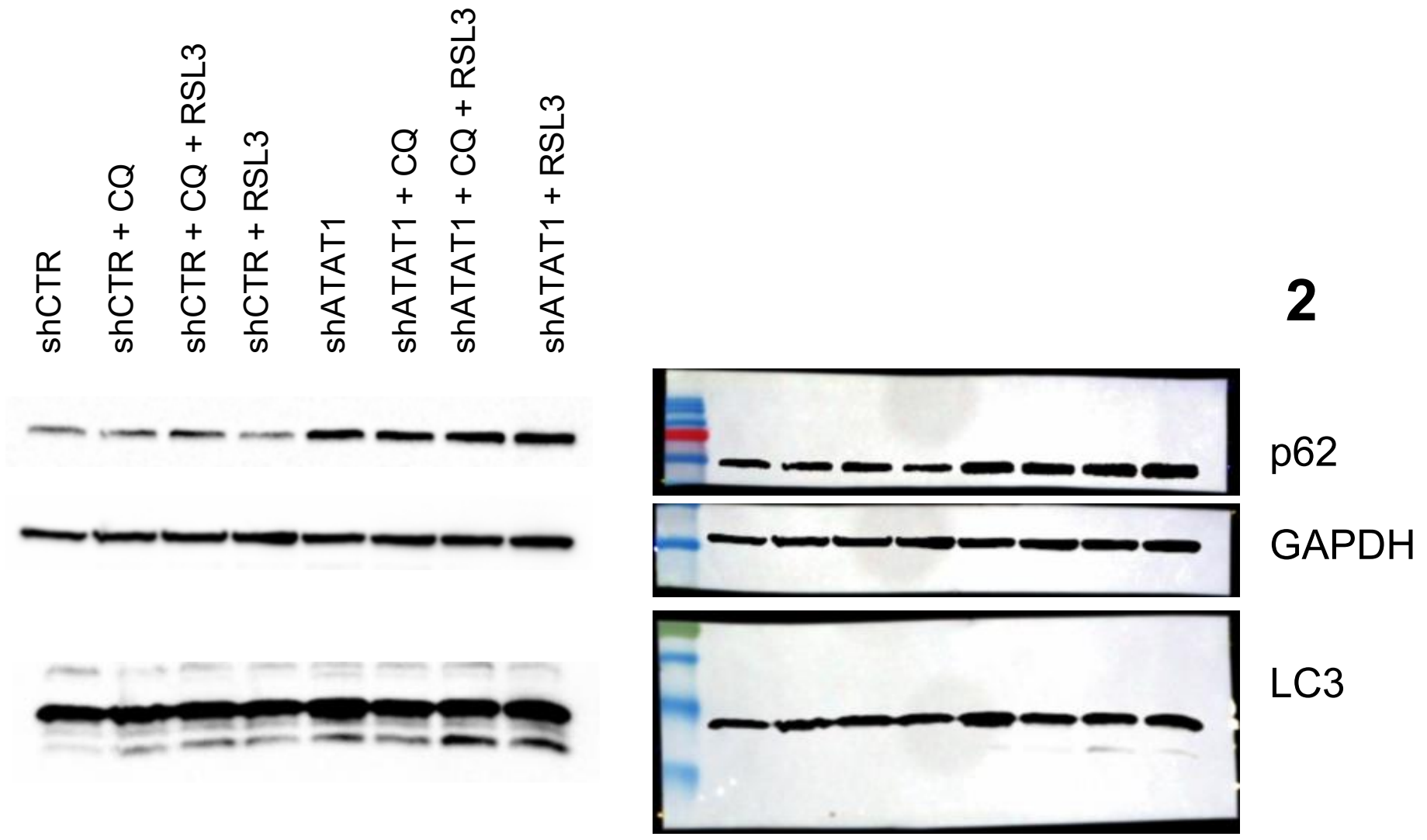

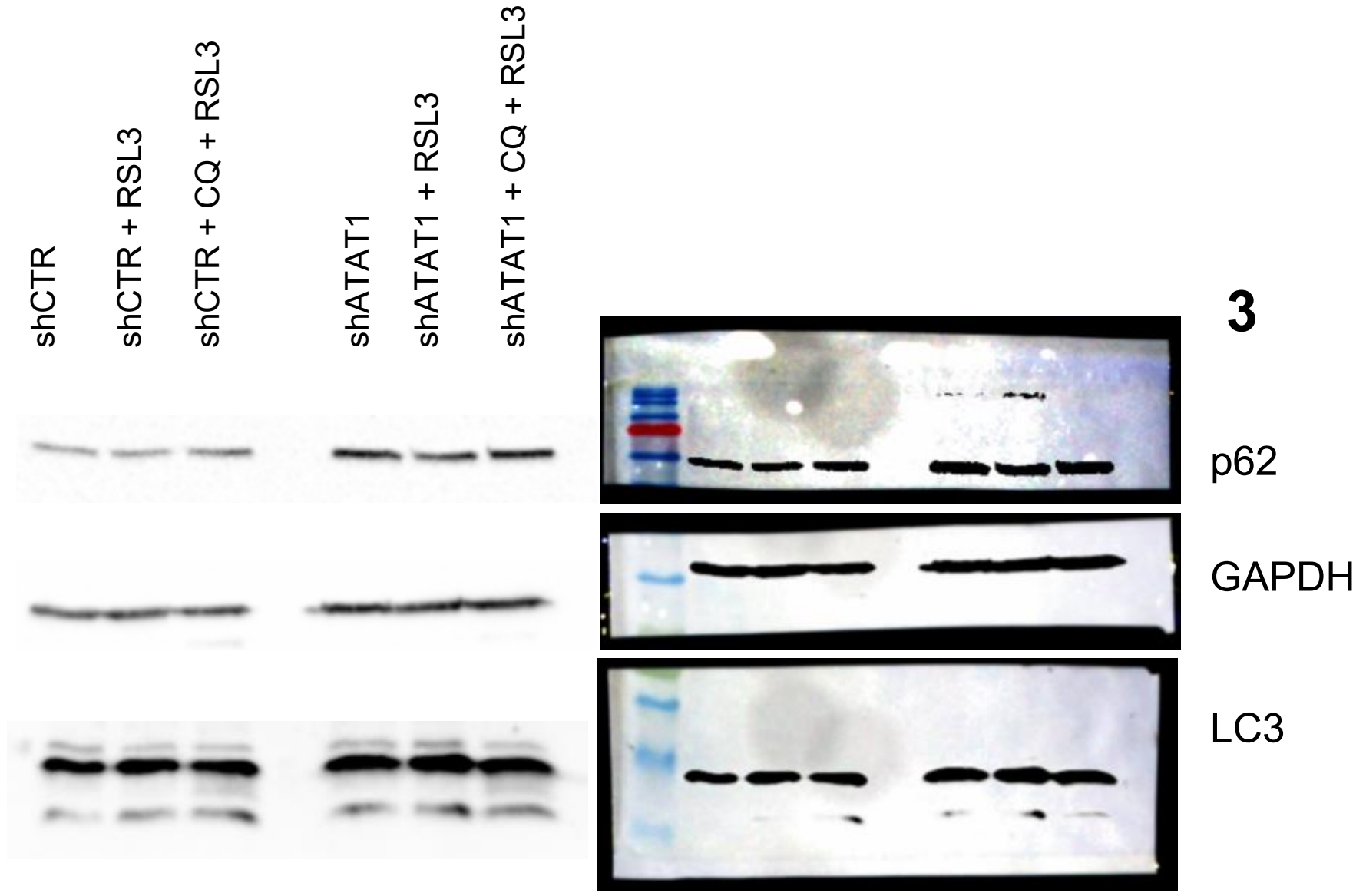

Figure 7C

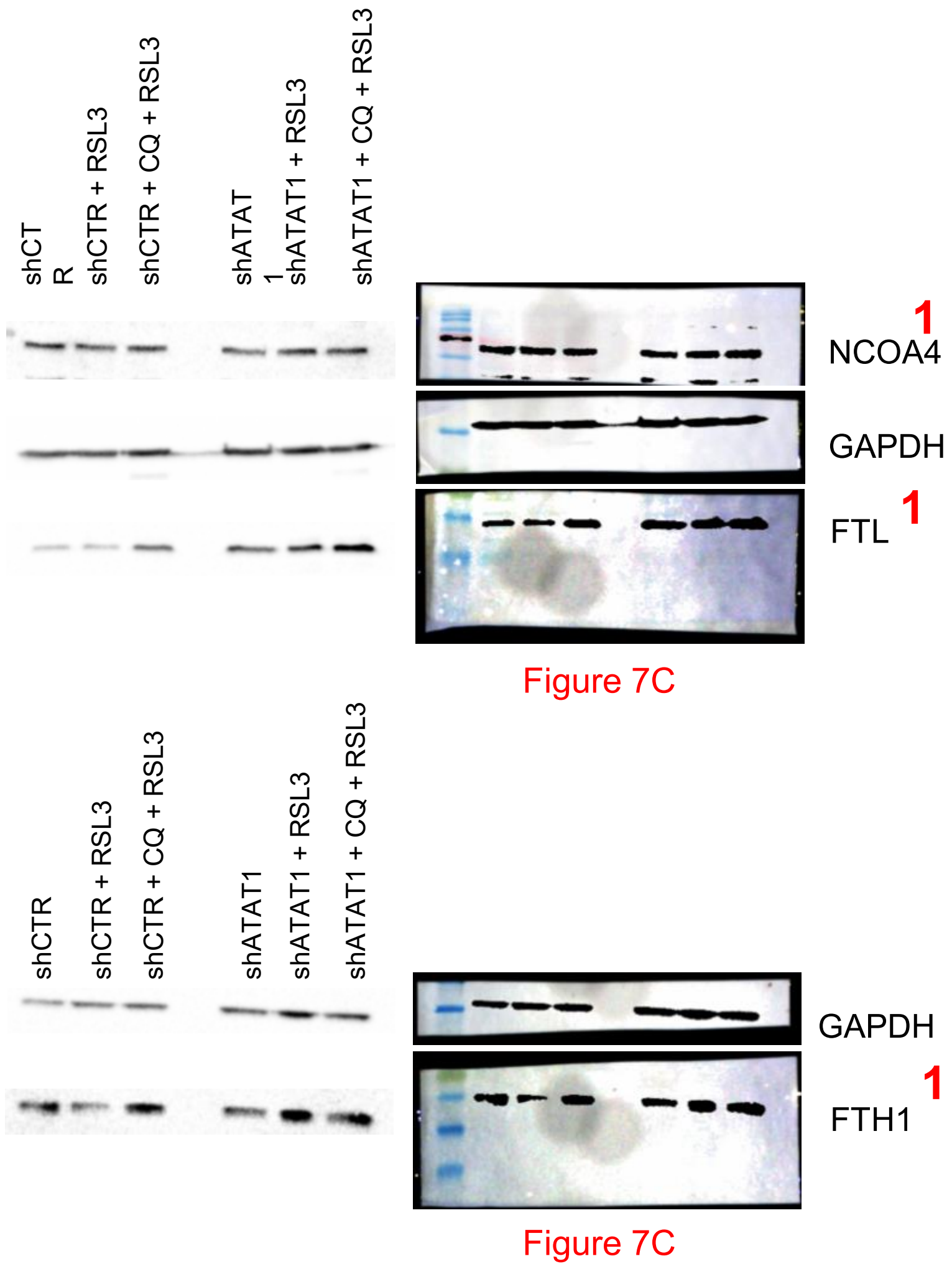

shCTR
shCTR + RSL3
shCTR + CQ + RSL3
shATAT1
shATAT1 + RSL3
shATAT1 + CQ + RSL3
NCOA4
1
GAPDH
FTL
1
Figure 7C
shCTR
shCTR + RSL3
shCTR + CQ + RSL3
shATAT1
shATAT1 + RSL3
shATAT1 + CQ + RSL3
GAPDH
FTH1
1
Figure 7C

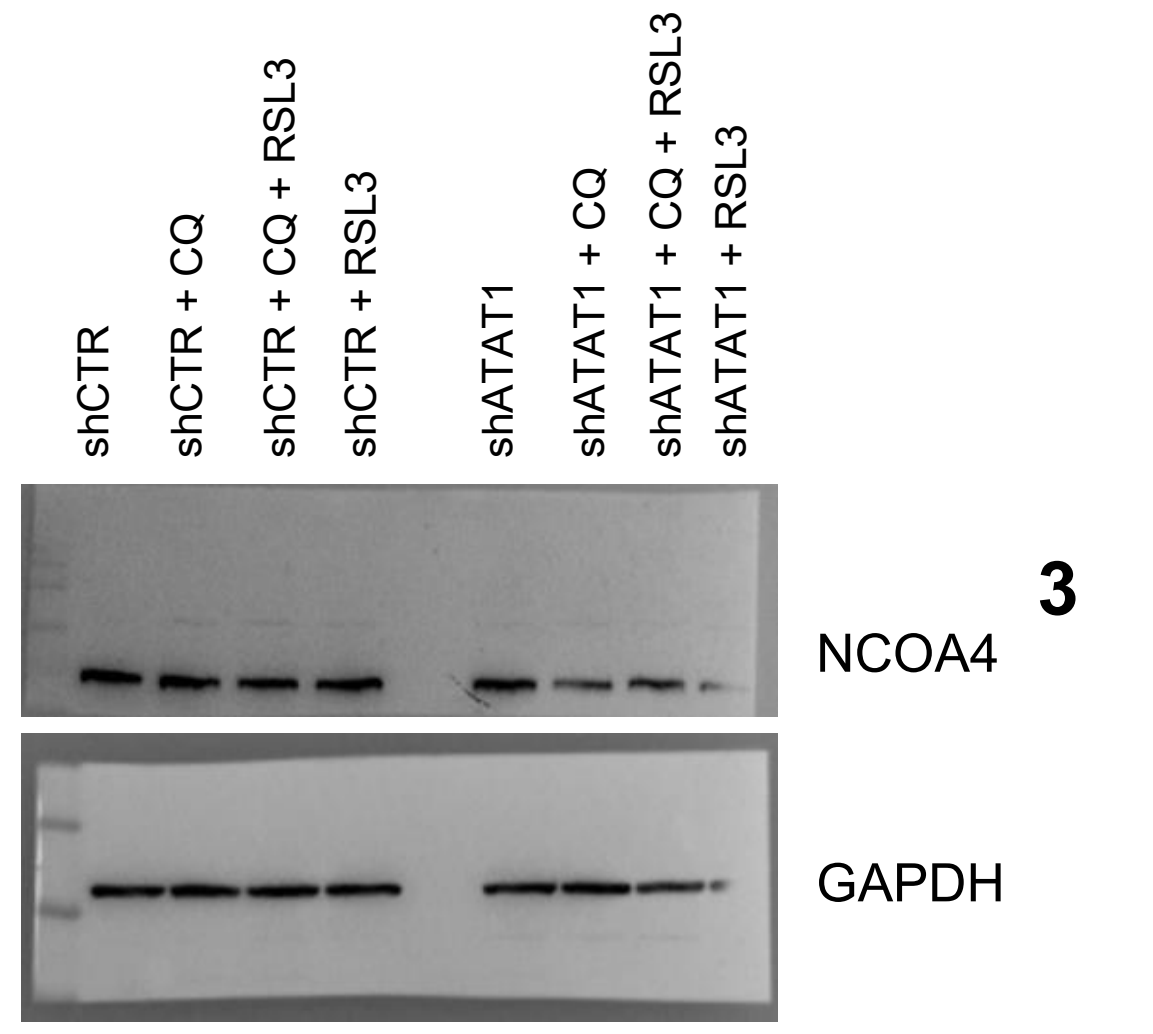

shCTR
shCTR + CQ
shCTR + CQ + RSL3
shCTR + RSL3
shATAT1
shATAT1 + CQ
shATAT1 + CQ + RSL3
shATAT1 + RSL3
NCOA4
3
GAPDH

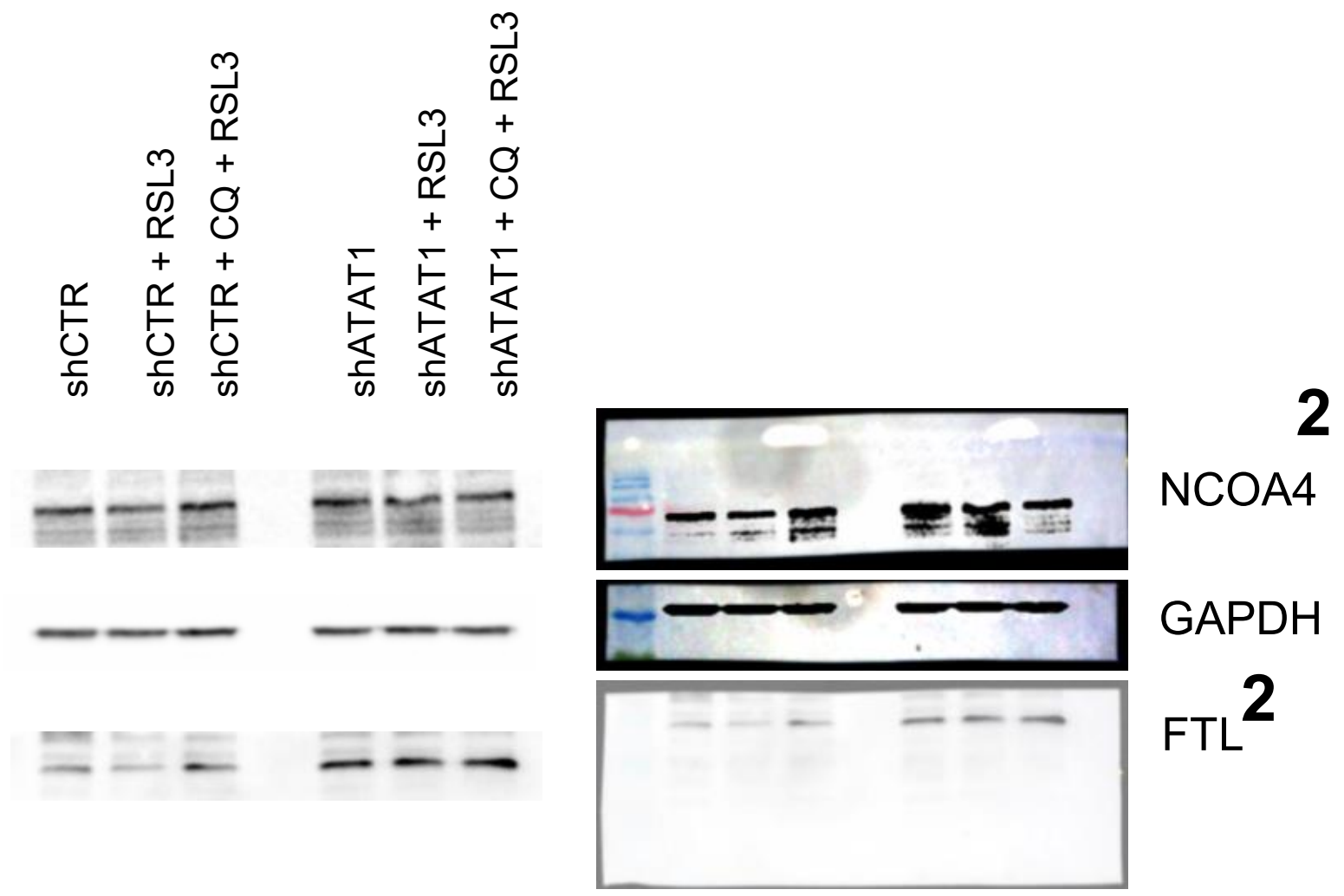

shCTR
shCTR + RSL3
shCTR + CQ + RSL3
shATAT1
shATAT1 + RSL3
shATAT1 + CQ + RSL3
NCOA4
2
GAPDH
FTL
2

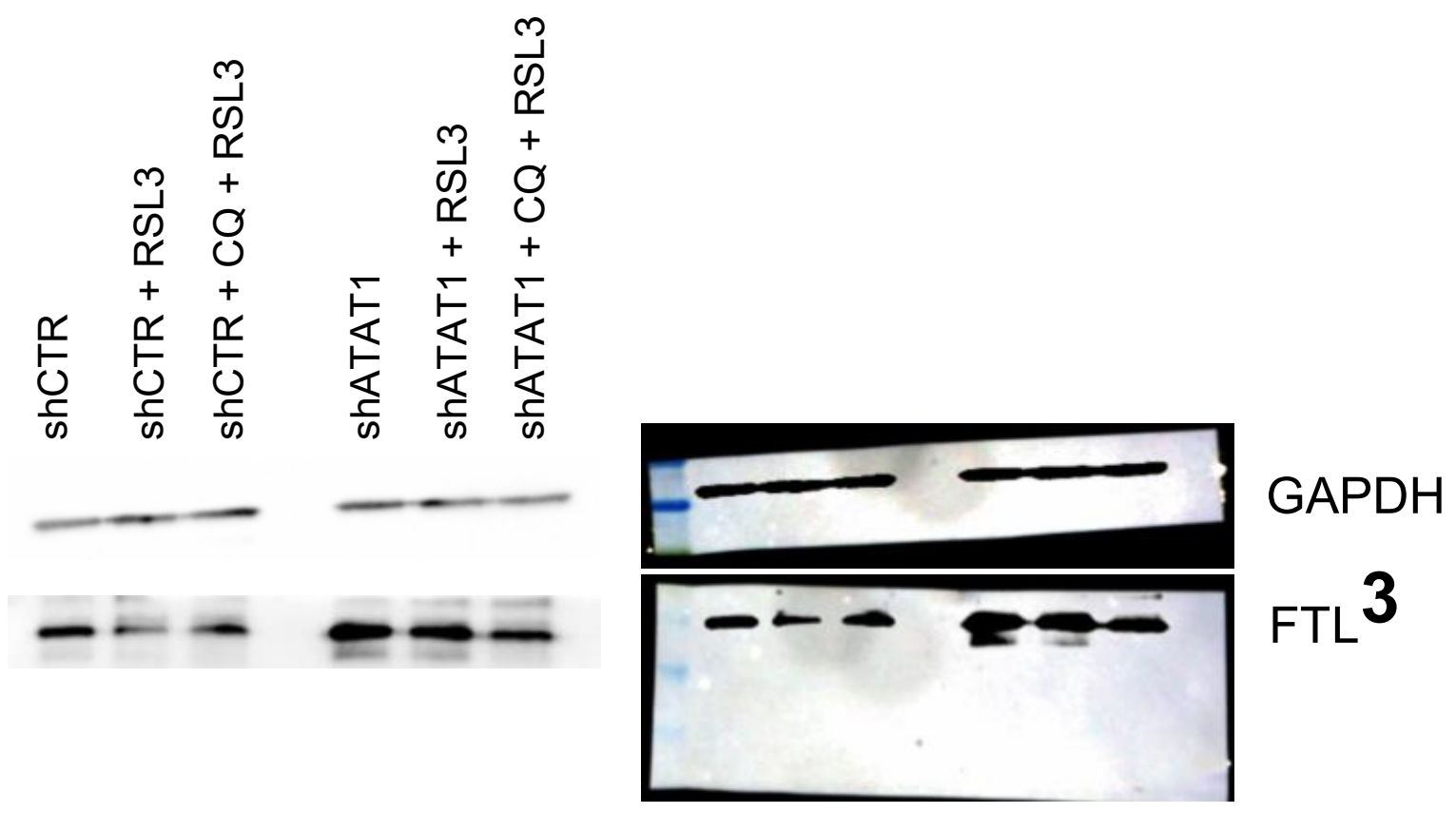

shCTR
shCTR + RSL3
shCTR + CQ + RSL3
shATAT1
shATAT1 + RSL3
shATAT1 + CQ + RSL3
GAPDH
FTL
3

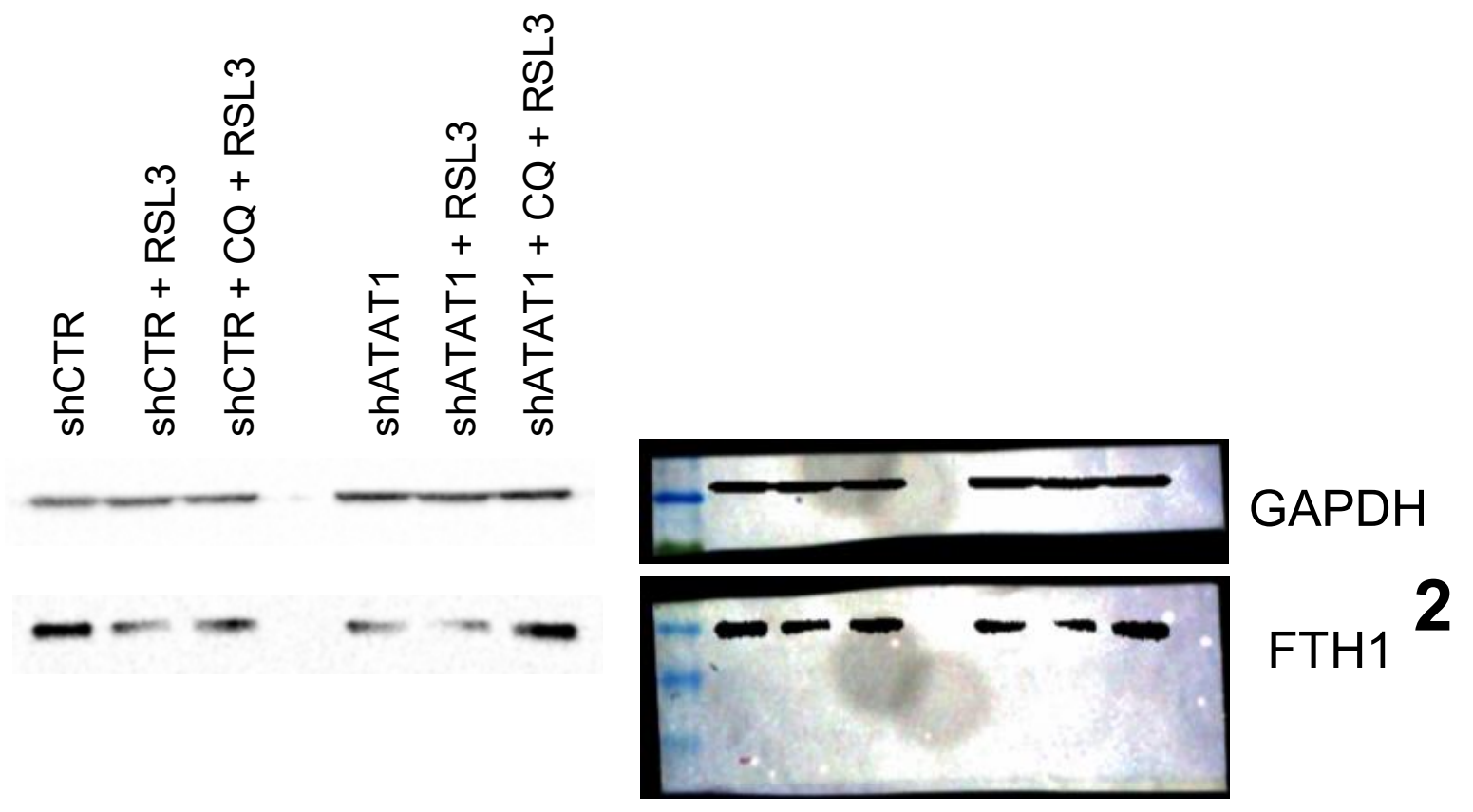

shCTR
shCTR + RSL3
shCTR + CQ + RSL3
shATAT1
shATAT1 + RSL3
shATAT1 + CQ + RSL3
GAPDH
FTH1
2

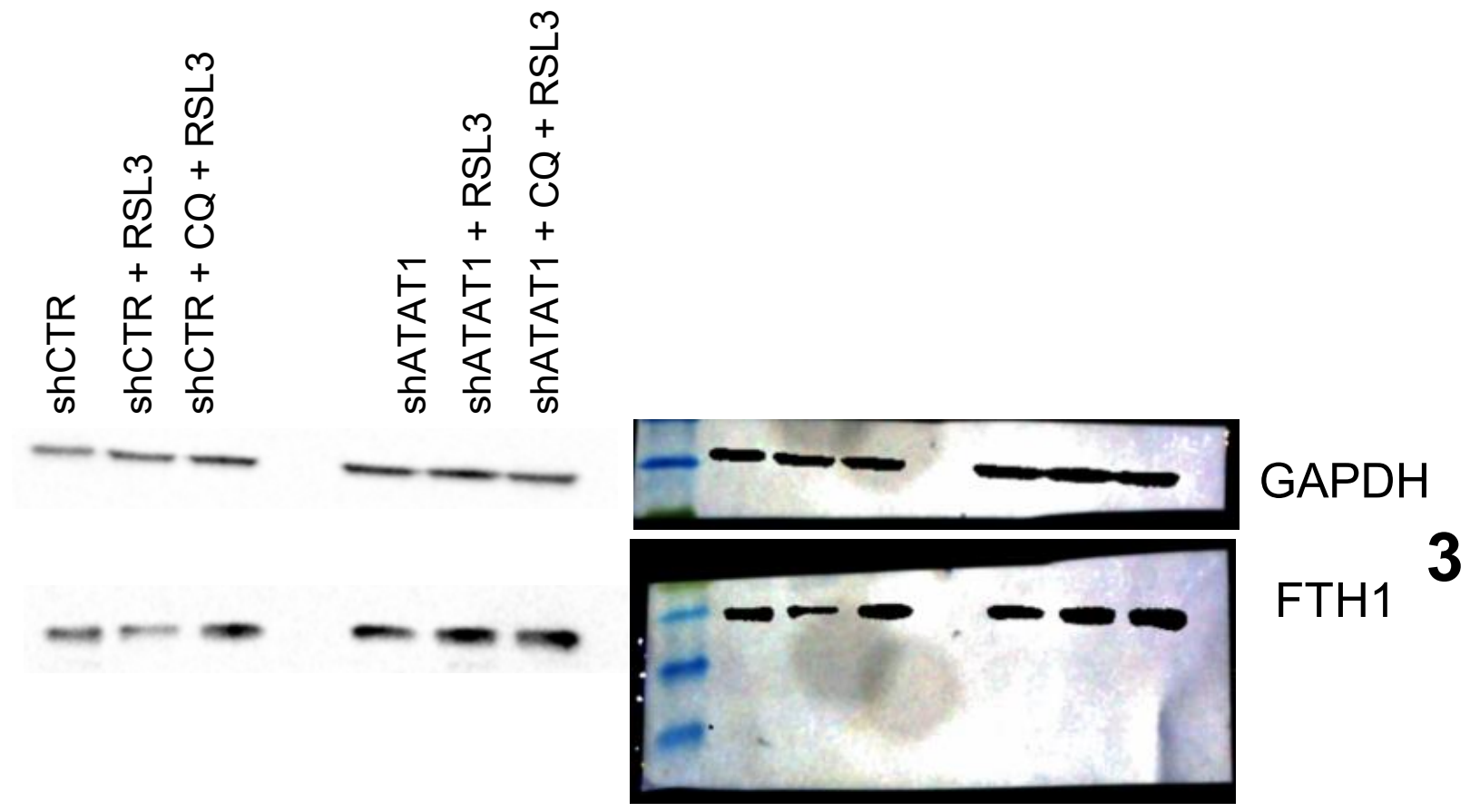

shCTR
shCTR + RSL3
shCTR + CQ + RSL3
shATAT1
shATAT1 + RSL3
shATAT1 + CQ + RSL3
GAPDH
FTH1
3

Figure S1A

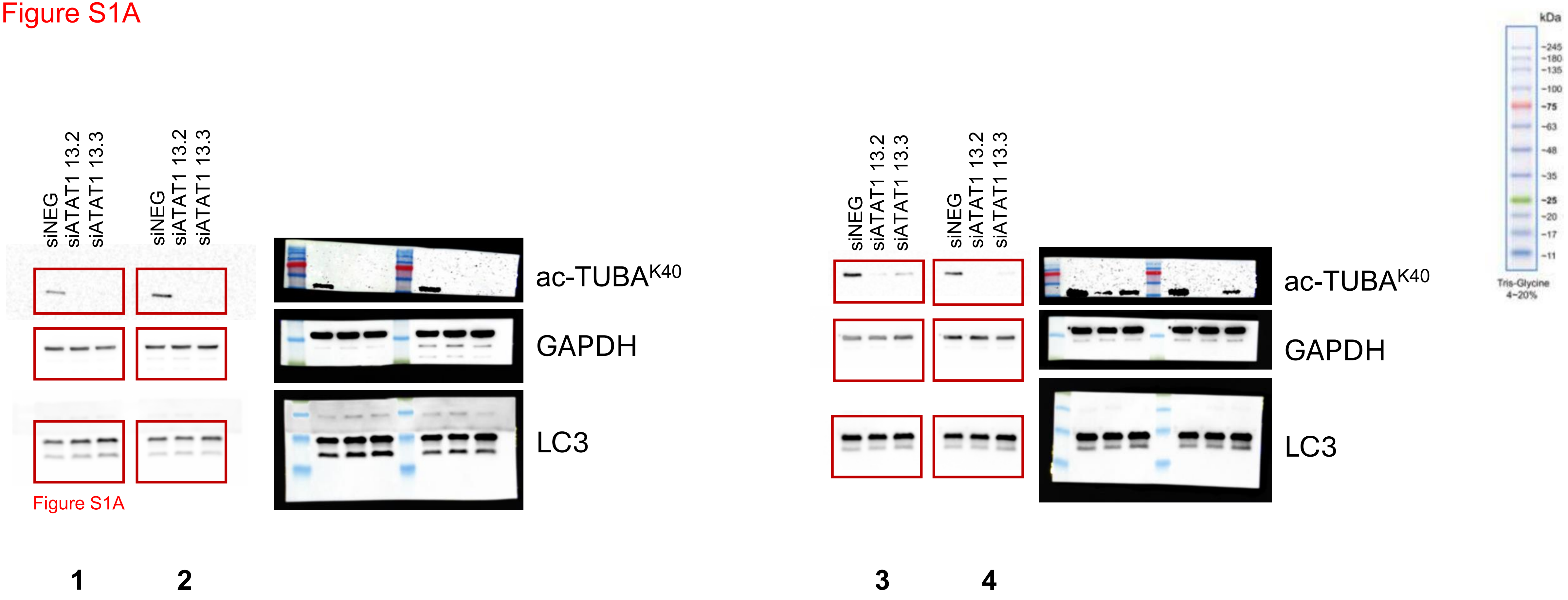
siNEG
siATAT1 13.2
siATAT1 13.3
ac-TUBAK40
GAPDH
LC3
Figure S1A
1
2
3
4
kDa
245
180
135
100
75
63
48
35
25
20
17
11
Tris-Glycine 4~20%

Figure S1C – H1299

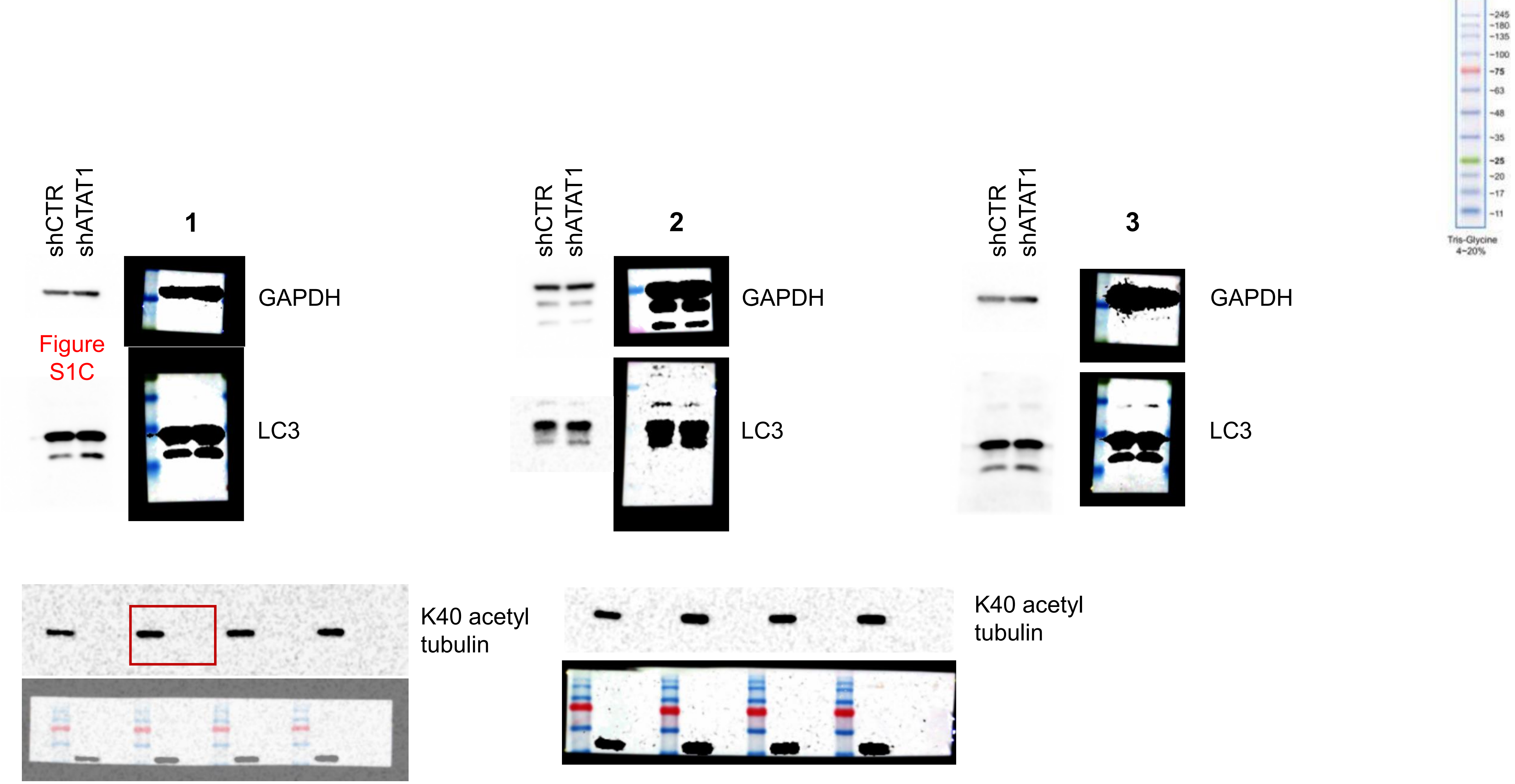

Figure S1C – A549

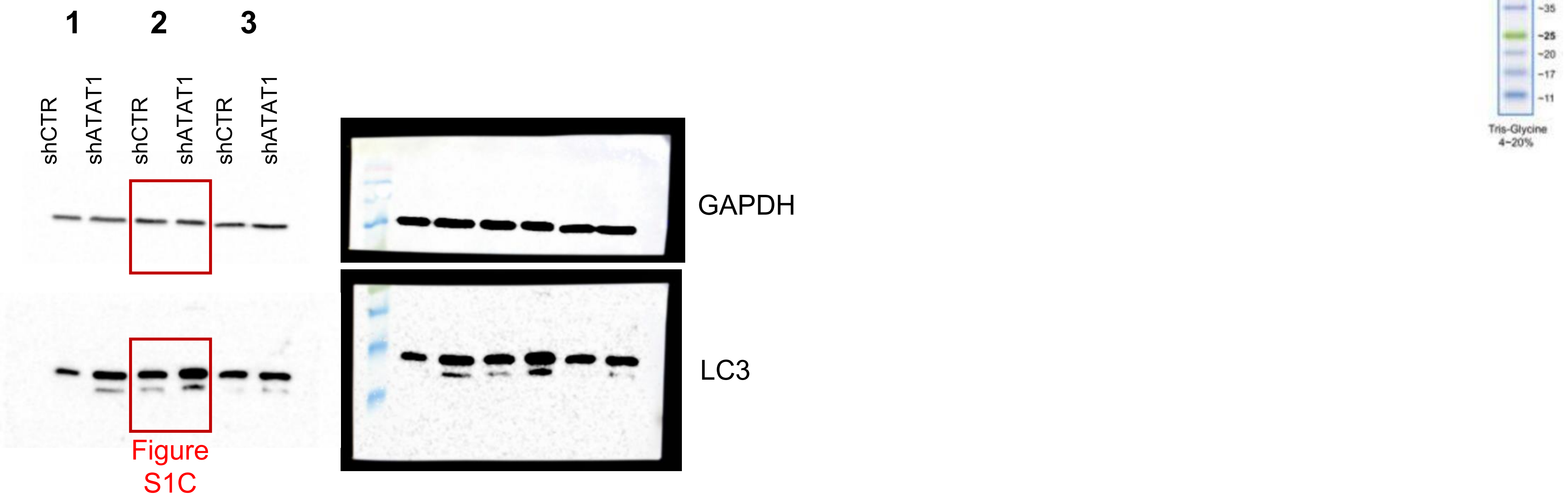

Figure S1H

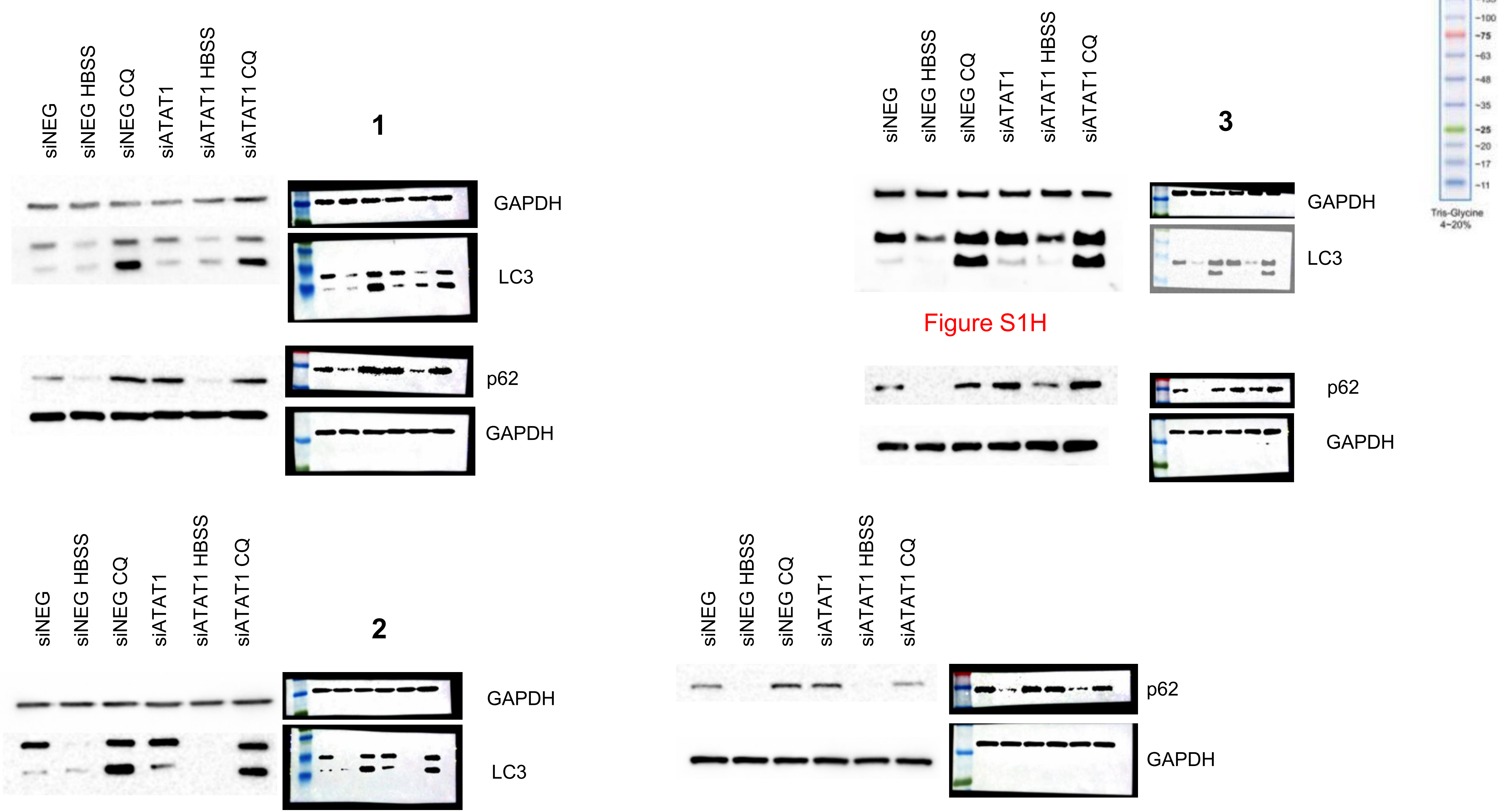

siNEG
siNEG HBSS
siNEG CQ
siATAT1
siATAT1 HBSS
siATAT1 CQ
1
GAPDH
LC3
p62
GAPDH
siNEG
siNEG HBSS
siNEG CQ
siATAT1
siATAT1 HBSS
siATAT1 CQ
3
GAPDH
LC3
Figure S1H
p62
GAPDH
kDa
245
180
135
100
75
63
48
35
25
20
17
11
Tris-Glycine
4~20%
siNEG
siNEG HBSS
siNEG CQ
siATAT1
siATAT1 HBSS
siATAT1 CQ
2
GAPDH
LC3
siNEG
siNEG HBSS
siNEG CQ
siATAT1
siATAT1 HBSS
siATAT1 CQ
p62
GAPDH

Figure S1I

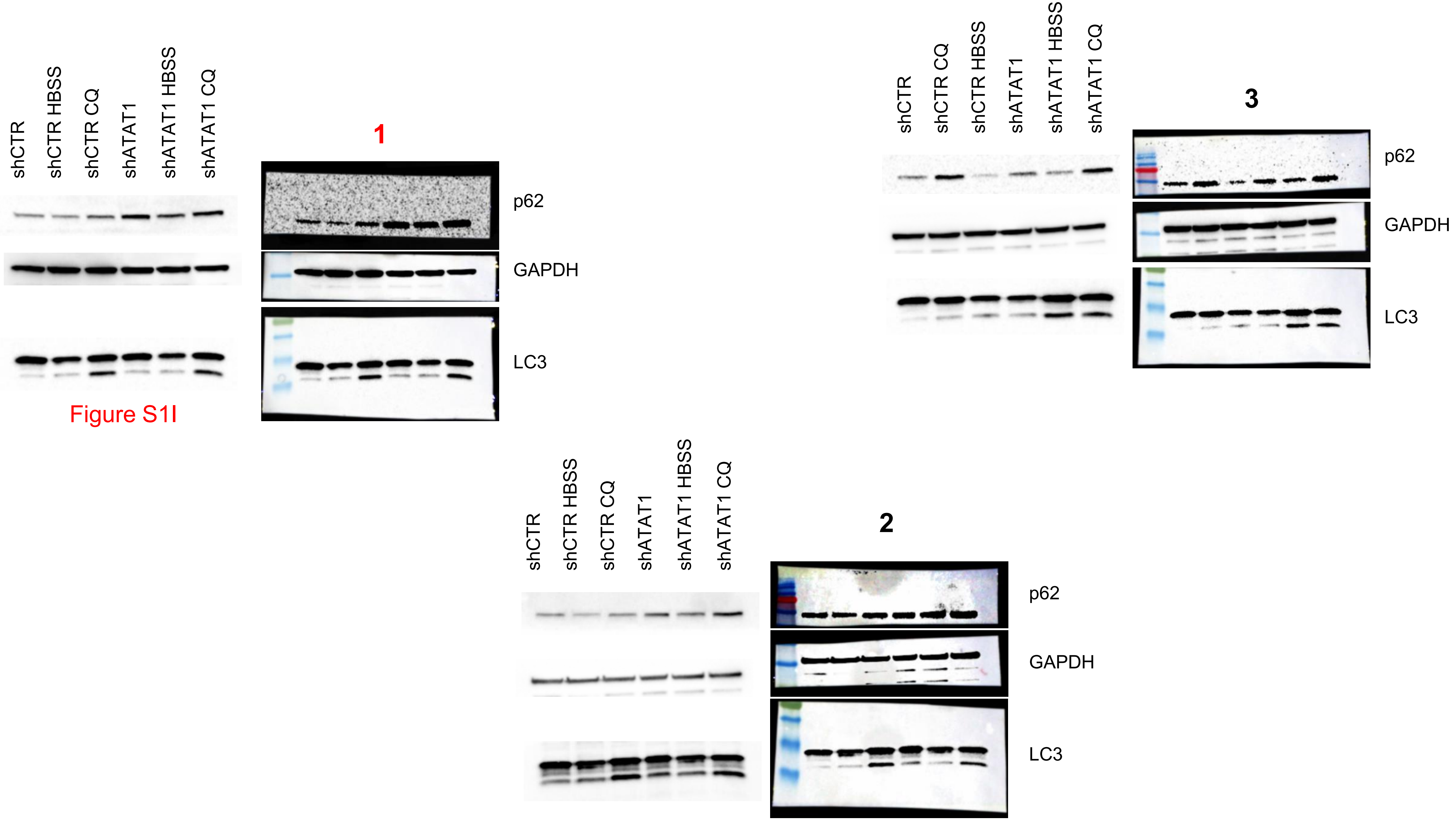

shCTR
shCTR HBSS
shCTR CQ
shATAT1
shATAT1 HBSS
shATAT1 CQ
1
p62
GAPDH
LC3
Figure S1I
shCTR
shCTR CQ
shCTR HBSS
shATAT1
shATAT1 HBSS
shATAT1 CQ
3
p62
GAPDH
LC3
shCTR
shCTR HBSS
shCTR CQ
shATAT1
shATAT1 HBSS
shATAT1 CQ
2
p62
GAPDH
LC3

Figure S2F

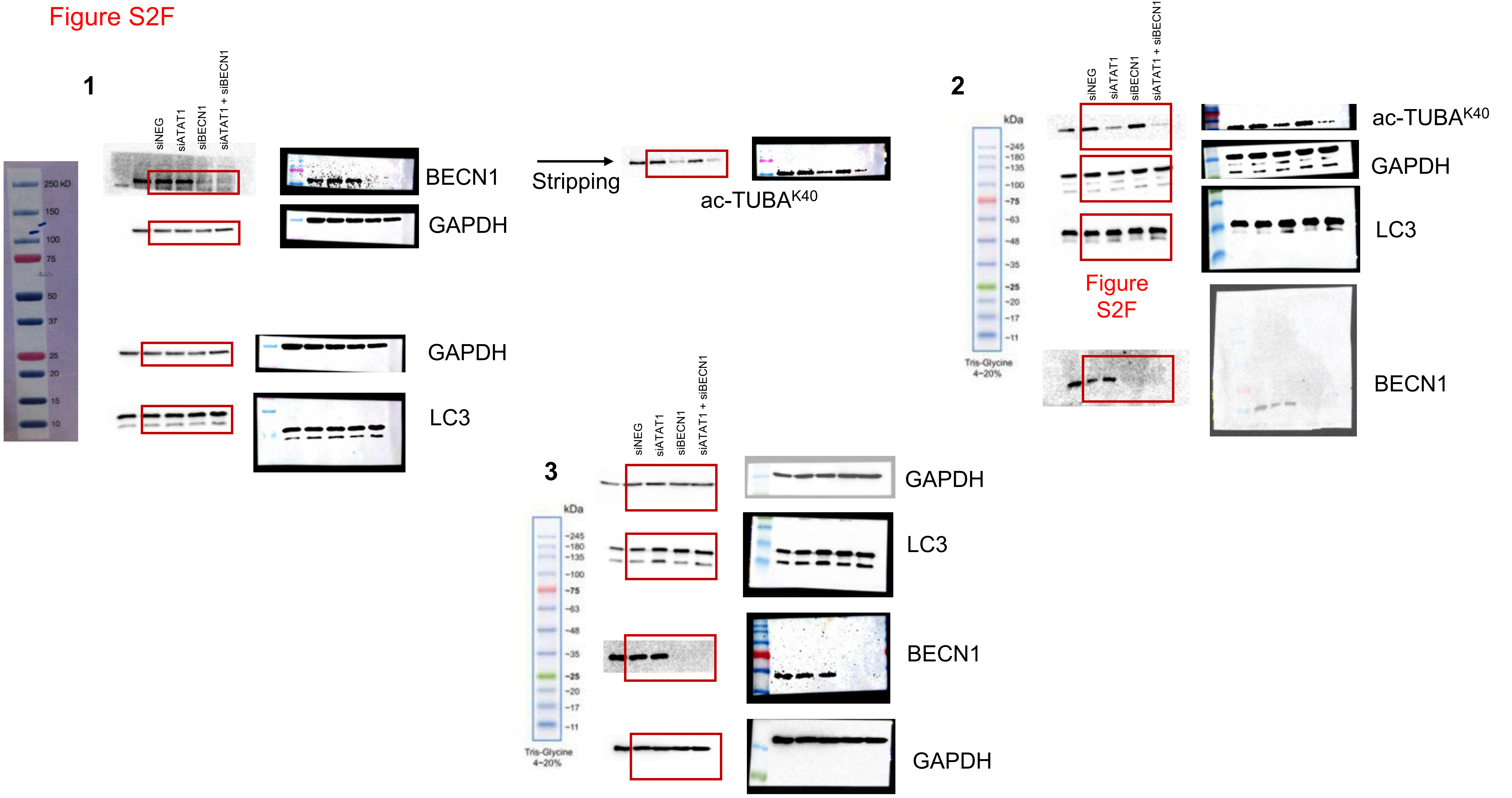

1
siNEG
siATAT1
siBECN1
siATAT1 + siBECN1
250 kD
150
100
75
50
37
25
20
15
10
BECN1
GAPDH
Stripping
ac-TUBAK40
GAPDH
LC3
2
siNEG
siATAT1
siBECN1
siATAT1 + siBECN1
kDa
245
180
135
100
75
63
48
35
25
20
17
11
Tris-Glycine 4~20%
ac-TUBAK40
GAPDH
LC3
Figure S2F
BECN1
3
siNEG
siATAT1
siBECN1
siATAT1 + siBECN1
kDa
Tris-Glycine 4~20%
GAPDH
LC3
BECN1
GAPDH

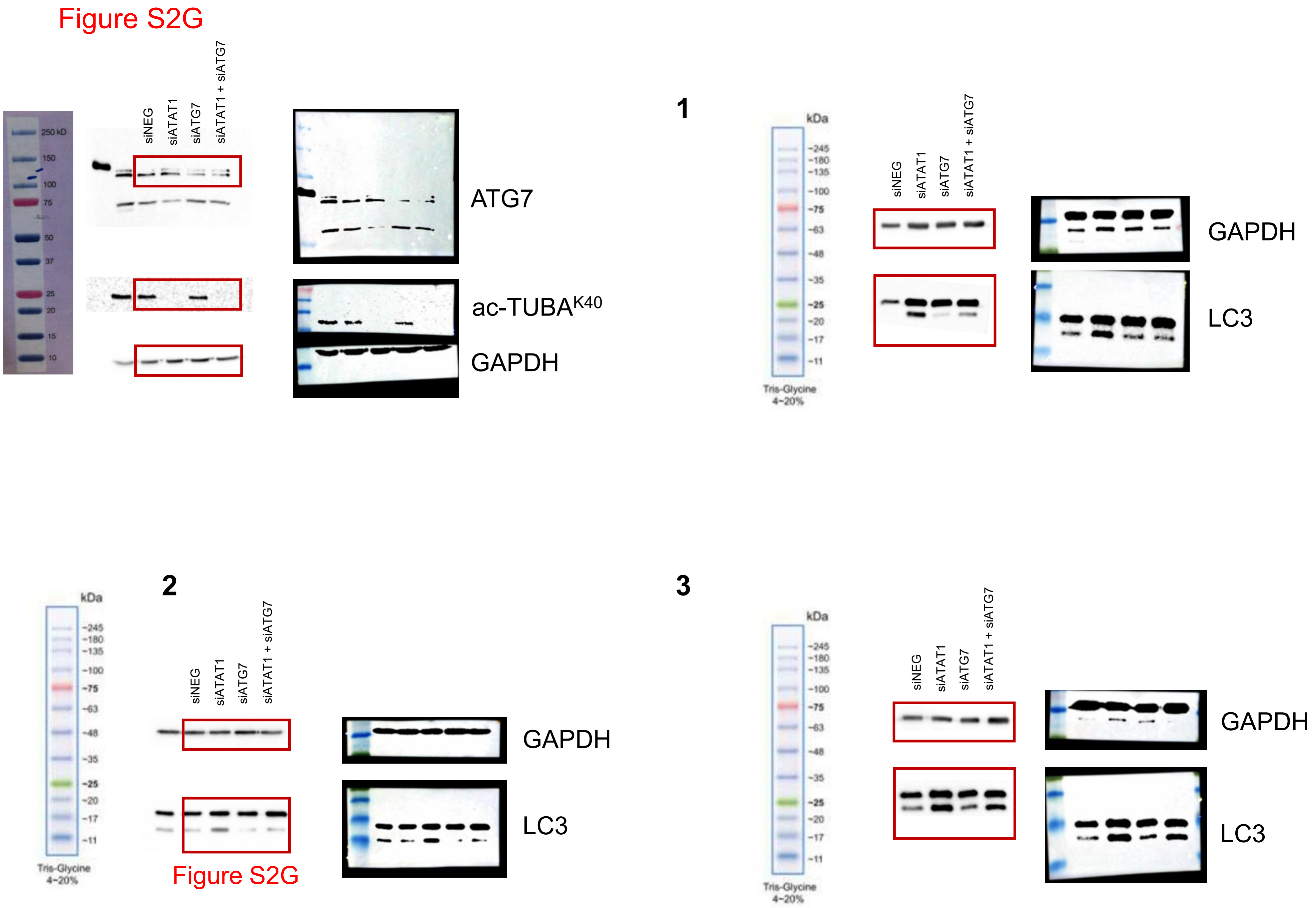
Figure S2G
250 kD
150
100
75
50
37
25
20
15
10
siNEG
siATAT1
siATG7
siATAT1 + siATG7
ATG7
ac-TUBAK40
GAPDH
1
kDa
245
180
135
100
75
63
48
35
25
20
17
11
Tris-Glycine 4~20%
GAPDH
LC3
2
3
Figure S2G

Figure S2M

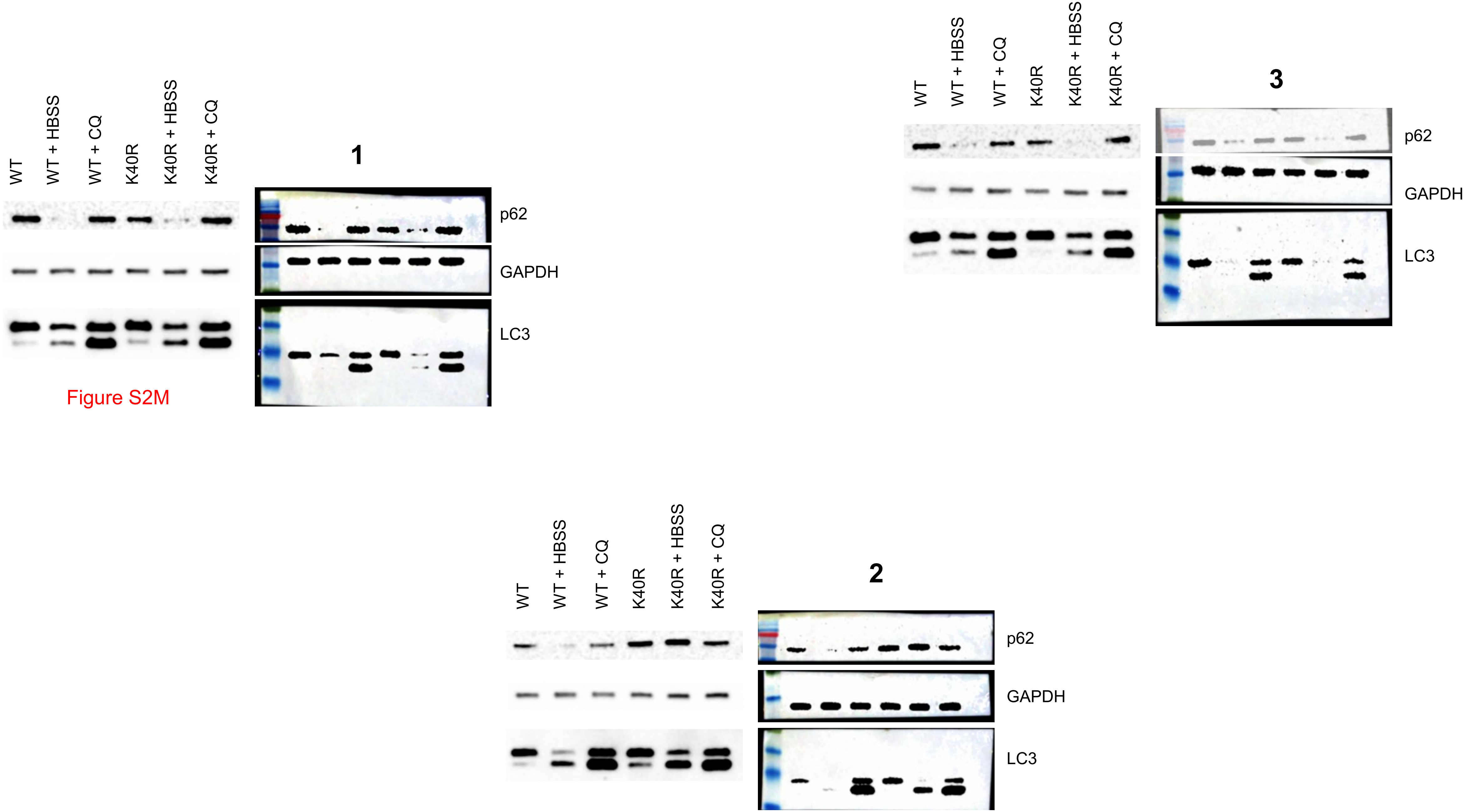

WT
WT + HBSS
WT + CQ
K40R
K40R + HBSS
K40R + CQ
1
p62
GAPDH
LC3
Figure S2M
WT
WT + HBSS
WT + CQ
K40R
K40R + HBSS
K40R + CQ
3
p62
GAPDH
LC3
WT
WT + HBSS
WT + CQ
K40R
K40R + HBSS
K40R + CQ
2
p62
GAPDH
LC3

Figure S3D siATAT1

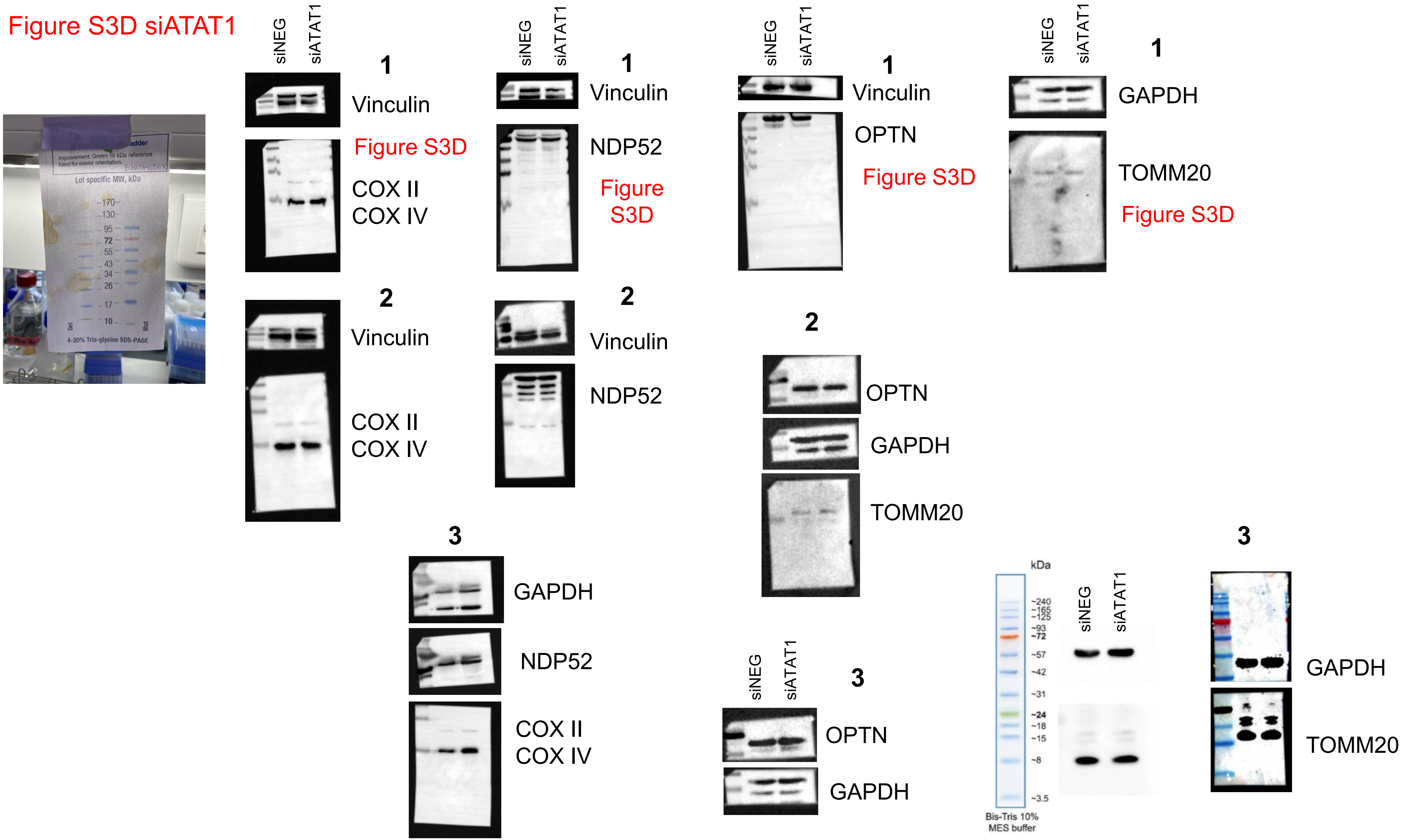

Figure S3D shATAT1

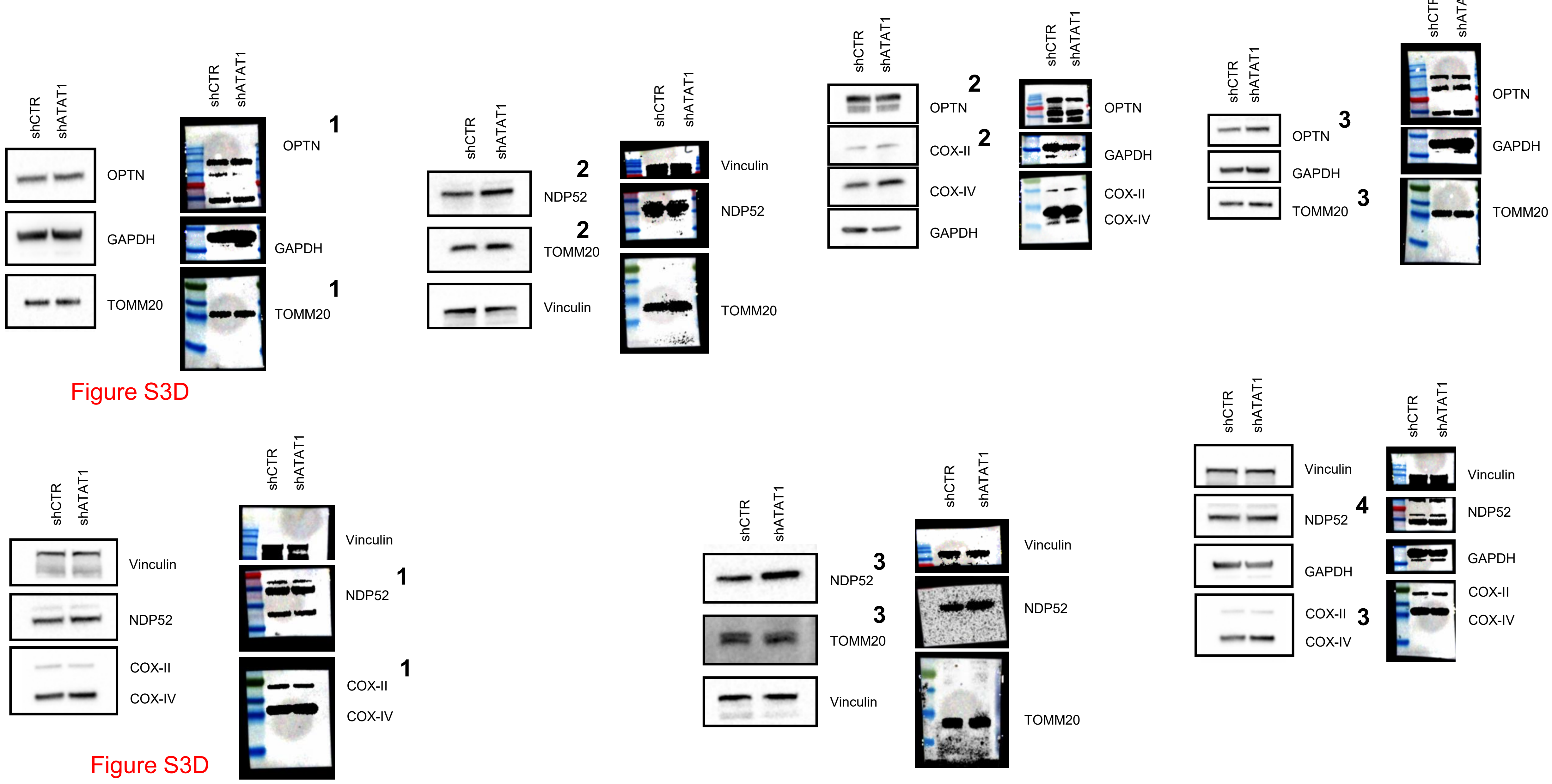

Supplement: Supplementary file 1 [file 11658_2026_964_MOESM6_ESM.pdf]
