# Supplementary material for: α-tubulin N-acetyltransferase 1 regulates NCOA4-mediated ferritinophagy and protects cancer cells from ferroptosis
Source: Cell Mol Biol Lett. 2026 Jun 12;31:134. doi: 10.1186/s11658-026-00964-2 (PMC13520228; doi:10.1186/s11658-026-00964-2)
Supplement: Supplementary file 2 [file 11658_2026_964_MOESM7_ESM.pdf]

Figure S1. (**A**) Immunoblot analysis of ac-TUBA^{K40} and LC3 conversion in H1299 cells transfected with siNEG or two different siRNA against ATAT1 (13.2 or 13.3). (**B**) qRT-PCR of ATAT1 mRNA in H1299 cells transfected as in (A). Data are presented as the ratio between ATAT1 mRNA and ACTNB (actin beta). Data are presented as mean ± SEM from one experiment conducted in triplicate and were analyzed using a Mann-Whitney t test. *P < 0.05. (**C**) Immunoblot analysis of ac-TUBA^{K40} and LC3 conversion in H1299 and A549 cells stably transfected with shRNA targeting ATAT1 (shATAT1) or with scramble shRNA (shCTR). (**D**) qRT-PCR of ATAT1 mRNA in H1299 stably transfected with shRNA targeting ATAT1 (shATAT1) or with scramble shRNA (shCTR). Data are presented as the ratio between ATAT1 mRNA and ACTNB (actin beta). Data are presented as mean ± SEM from four independent experiments and were analyzed using a Mann-Whitney t test. *P < 0.05. (**E**) Representative images of H1299 cells transfected with siNEG or siATAT1, untreated (NT) or treated with 100 nM Bafilomycin A1 (Baf) or 25 μM Chloroquine (CQ) and incubated with DQ-BSA Red according to manifacturer's instructions. Cells were counterstained with DAPI (blue). Scale bars = 10 μm. (**F,G**) Densitometric analysis of LC3 and SQSTM1/p62 in H1299 cells transfected with siNEG or siATAT1 alone or in combination with HBSS or 25 μM CQ. Data are presented as mean ± SEM from at least three independent experiments, shown as fold change (FC), and were analyzed using a Kruskal-Wallis test followed an uncorrected Dunn's post-hoc test. *P < 0.05, ***P < 0.001. (**H**) Immunoblot analysis of LC3 and SQSTM1/p62 in HeLa cells transfected with siNEG or siATAT1 alone or in combination with HBSS or 25 μM CQ. Numbers on immunoblots report the densitometric values of band intensity normalized over GAPDH. (**I**) Immunoblot analysis of LC3 and SQSTM1/p62 in H1299 cells stably transfected with shRNA targeting ATAT1 (shATAT1) or with scramble shRNA (shCTR) alone or in combination with HBSS or 25 μM CQ.

Figure S2. (**A-E**) Densitometric analysis of SQSTM1/p62, NBR1, p-AMPK$^{(T172)}$, p-ULK1$^{(Ser555)}$, p-mTOR$^{(Ser2448)}$ in H1299 cells transfected with siNEG or siATAT1. Densitometric data are presented as mean ± SEM from at least three independent experiments, shown as fold change (FC), and were

analyzed using a Mann-Whitney test. *P < 0.05, **P < 0.01, ***P < 0.001. (**F,G**) Representative immunoblot analysis of LC3, ATG7, BECN1 and ac-TUBA^{K40} in H1299 cells transfected with a siNEG or a siRNA against BECN1 (siBECN1) or against ATG7 (siATG7) for 72 h, in combination or not with siATAT1 for the last 48 h. Numbers on immunoblots report the densitometric values of band intensity normalized over GAPDH. (**H–L**) Densitometric analysis of LC3, SQSTM1/p62, and p-AMPK$^{(T172)}$ in H1299 cells transfected with siNEG or siATAT1 and treated or not with 5 mM NAC for 42h or 5 mM H2O2 for 30 min. Data are presented as mean ± SEM from at least three independent experiments, shown as fold change (FC), and were analyzed using a Kruskal-Wallis test followed an uncorrected Dunn's post-hoc test. *P < 0.05. (**M**) Immunoblot analysis of LC3 and SQSTM1/p62 in H1299 cells expressing WT TUBA or the mutant form TUBA^{K40R} alone or in combination with HBSS or 25 μM CQ. (**N**) Densitometric analysis of p-AMPK$^{(Thr1172)}$ in H1299 cells expressing WT TUBA or the mutant form TUBA^{K40R}. Data are presented as mean ± SEM from at least three independent experiments.

Figure S3. (**A**) Representative images and (**B**) quantification of the percentage of cells with Parkin (PRKN) spots recruited on mitochondria in H1299 cells stably transfected with shRNA targeting ATAT1 (shATAT1) or with scramble shRNA (shCTR) alone or in combination with FCCP for 6 h. Data are presented as mean ± SEM from three independent experiments and were analyzed using an ordinary One-Way ANOVA followed by a Fisher's LSD post-hoc test. ****P < 0.0001. (**C**) Cytofluorimetric quantification of the ratio between the number of cells excited at 620 nm (pH4) and excited at 440 nm (pH7) in Mito-Keima H1299 cells transfected with siNEG or siATAT1 alone or in combination with FCCP for 6 h. Data are presented as mean ± SEM from three independent experiments and were analyzed using a Kruskal-Wallis test followed by Dunn's post-hoc test. *P < 0.05. (**D**) Immunoblot analysis of NDP52, OPTN, COX II, COX IV and TOMM20 protein levels in H1299 cell transiently or stably silenced for ATAT1. (**E**) Quantification of mitochondrial DNA in H1299 cells transiently or (**F**) stably silenced for ATAT1 expression. Data are presented as the ratio

between mtDNA content and total genomic DNA. Data are presented as mean ± SEM from at least two independent experiments, shown as FC and were analyzed using a non-parametric Mann-Whitney t test. *P < 0.05. (**G-M**) Densitometric analysis of NCOA4, TFRC, LC3-II, FTL and FTH protein levels in H1299 cells transfected with siNEG or siATAT1 treated or not with 10 μM Chloroquine (CQ). Data are presented as mean ± SEM from at least two independent experiments, shown as fold change (FC) and were analyzed using Kruskal-Wallis test followed by Dunn's post-hoc test. *P < 0.05, **P < 0.01.

Figure S4. (**A**) Analysis of cell viability evaluated by MTT assay of H1299 and A549 cells stably transfected with shRNA targeting ATAT1 (shATAT1) or with scramble shRNA (shCTR) treated with increasing concentration of RSL3 for 24 h. (**B**) Analysis of cell viability evaluated by MTT assay of H1299 cells stably transfected with shRNA targeting ATAT1 (shATAT1) or with scramble shRNA (shCTR) treated with increasing concentration of Erastin for 24 h. (**C**) Quantification of the percentage of ANXA5 positive A549 cells stably transfected with shRNA targeting ATAT1 (shATAT1) or with scramble shRNA (shCTR) alone or in combination with 1 μM RSL3, 1 μM Ferrostatin-1, or with a combination of the two drugs. Data are presented as mean ± SEM from three independent experiments and were analyzed using an ordinary One-Way ANOVA test followed by uncorrected Fisher's LSD post-hoc test. (**D**) Quantification of the percentage of oxidized Bodipy C11 positive cells in A549 cells treated as in (C). Data are presented as mean ± SEM from at least three independent experiments and were analyzed using an ordinary One-Way ANOVA test followed by uncorrected Fisher's LSD post-hoc test. *P <0.05. **P < 0.01, ***P < 0.001, ****P < 0.0001. (**E**) Quantification of the percentage of SubG1 peak in n H1299 cells expressing WT TUBA or the mutant form $TUBA^{K40R}$ alone or with 50 nM RSL3. Data are presented as mean ± SEM from two independent experiments.

Figure S5. Densitometric analysis of p62, LC3-II, NCOA4, FTL and FTH protein levels in H1299 cells transfected with siNEG or siATAT1 treated or not with RSL3 50nM alone or in combination with 10 μM Chloroquine (CQ). Data are presented as mean ± SEM from at least two independent experiments, shown as fold change (FC) and were analyzed using a Kruskal-Wallis test followed by Dunn's post-hoc test. $^{*}P < 0.05$.

Figure S6. Uncropped original western blots

Video 1S. Representative video of mRFP-EGFP-LC3 H1299 cells transfected with siNEG. Cells were recorded from 24 h post-transfection. Images were acquired at 20 min intervals for 50 h.

Video2S. Representative video of mRFP-EGFP-LC3 H1299 cells transfected with siATAT1. Cells were recorded from 24 h post-transfection. Images were acquired at 20 min intervals for 50 h.
